# A Conjugated Microporous Polymer for Palladium‐Free, Visible Light‐Promoted Photocatalytic Stille‐Type Coupling Reactions

**DOI:** 10.1002/advs.201700101

**Published:** 2017-05-22

**Authors:** Saman Ghasimi, Simon A. Bretschneider, Wei Huang, Katharina Landfester, Kai A. I. Zhang

**Affiliations:** ^1^ Max Planck Institute for Polymer Research Ackermannweg 10 Mainz 55128 Germany

**Keywords:** conjugated microporous polymers, destannylation, palladium‐free, photocatalysis, Stille coupling

## Abstract

The Stille coupling reaction is a versatile method to mainly form aromatic C—C bonds. However, up to now, the use of palladium catalysts is necessary. Here, a palladium‐free and photocatalytic Stille‐type coupling reaction of aryl iodides and aryl stannanes catalyzing a conjugated microporous polymer‐based phototcatalyst under visible light irradiation at room temperature is reported. The novel coupling reaction mechanism occurs between the photogenerated aryl radical under oxidative destannylation of the aryl stannane, and the electron‐activated aryl iodide, resulting into the aromatic C—C bond formation reaction. The visible light‐promoted Stille‐type coupling reaction using the polymer‐based pure organic photocatalyst offers a simple, sustainable, and more economic synthetic pathway toward palladium‐free aromatic C—C bond formation.

The Stille cross‐coupling reaction is a highly versatile tool for carbon–carbon bond formations from aryl halides and aryl stannanes.^1^ The major requirement for the Stille coupling, however, is the necessary use of palladium complexes as catalysts (Equation (1)). The search for pure organic and palladium‐free alternative catalysts still remains a huge challenge for the organic and material chemists.

Traditional Stille coupling

(1) $${\mathrm{Ar}}^{1}\;-\;X\;+\;R_{3}\mathrm{Sn}\;-\;{\mathrm{Ar}}^{2}\;\overset{\mathrm{Pd}(0)}{\underset{\mathrm{elevated}\;\mathrm{temperature}}{\to }}\;{\mathrm{Ar}}^{1}\;-\;{\mathrm{Ar}}^{2}$$

This work

(2) $${\mathrm{Ar}}^{1}-X+R_{3}\mathrm{Sn}-{\mathrm{Ar}}^{2}\overset{\mathrm{organic}\;\mathrm{catalyst}}{\underset{\mathrm{with}\;\mathrm{light},\;\mathrm{RT}}{\to }}{\mathrm{Ar}}^{1}-{\mathrm{Ar}}^{2}$$

Ar: aryl; X: halide; R: alkyl

Unlike the thermally activated reaction mechanism of the Stille coupling via various palladium complexes as intermediates, the radical‐based bond formation pathway offers an interesting alternative. Especially room temperature (RT)‐active, visible light‐generated photocatalytic alkylation^2^ or arylation^3^, ^4^, ^5^ reactions have been demonstrated as a versatile tool for C—C bond formations.^6^, ^7^ In the past, a number of visible light‐active catalytic systems have been developed. Among them, metal‐free photocatalytic systems^8^, ^9^ offer a more sustainable and environmentally friendly alternative compared to the traditional transition metal complexes such as Ru(bpy)_3_Cl_2_ and *fac*‐Ir(ppy)_3_,^9^, ^10^ or recently reported common metal‐based photocatalysts such as copper.^11^ Among the recently developed nonmetal photocatalysts, conjugated small molecule^4^, ^12^, ^13^ and especially macromolecular^8^, ^14^ systems have been employed as efficient photocatalyts for visible light‐promoted photocatalytic reactions. As a recently emerging macromolecular photocatalytic system, conjugated microporous polymers, which combine semiconductor property and high interfacial properties, have demonstrated their versatile utilization for visible light‐promoted photoredox reactions. Given their tunable electronic and optical properties via molecular design or morphology engineering, we envision that the further development of conjugated microporous polymers as photocatalysts could lead to the realization of challenging reactions such as noble meal‐free aromatic C—C bond formation.

Here, we report on a palladium catalyst‐free and photocatalytic Stille‐type aromatic C—C bond formation pathway of aryl iodides and aryl stannanes using conjugated organic phototcatalysts under visible light irradiation at room temperature. The novel coupling reaction mechanism occurred between the photogenerated aryl radical under oxidative destannylation of the aryl stannane, and the electron‐activated aryl iodide, resulting into the aromatic C—C bond formation reaction. Further studies using radical trapping agent were conducted to reveal the mechanistic insight of the photocatalytic Stille‐type coupling reaction.

In this study, a conjugated azulene‐containing microporous polymer network was chosen as the visible light‐active and pure organic photocatalyst. The polymer P‐Az‐B was obtained via Suzuki cross‐coupling reaction of 1,3‐dibromoazulene with 1,3,5‐phenyltriboronic acid tris(pinacol) ester with 1,3‐dibromoazulene (**Figure** **1**a). To note, an optimized synthetic route was developed. Here, the comomoner 1,3‐dibromoazulene was successively added to the reaction mixture in order obtain high porosity of the polymer. The synthetic details and characterization data are described in the Supporting Information.

**Figure 1:**
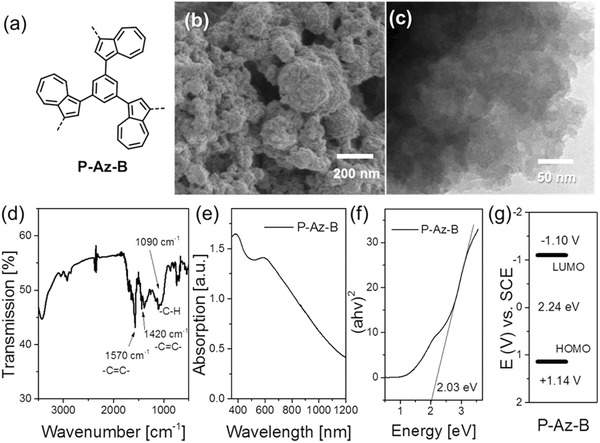
a) Structure, b) SEM and c) TEM image, d) FTIR, e) UV–vis DR spectra, f) Tauc plot, and g) HOMO and LUMO band positions of P‐Az‐B.

P‐Az‐B was insoluble in all common organic solvents tested. The scanning electron microscopy (SEM) and transmission electron microscopy (TEM) of P‐Az‐B showed a fused porous particle‐like morphology (Figure 1b). The Brunauer–Emmett–Teller (BET) surface area of P‐Az‐B was found to be 292 m^2^ g^−1^, achieving a significant increase of ≈17‐fold compared to the same polymer via unmodified bulk synthesis (*S*_BET_ = 17 m^2^ g^−1^, Table S1, Supporting Information). Additionally, using different solvents was not found to significantly affect the BET surface area of P‐Az‐B (Table S1, Supporting Information). ^13^C cross polarization magic angle spinning (CP/MAS) NMR spectroscopy (Figure S1, Supporting Information) showed characteristic peaks at 123, 130, 136, and 137 ppm, which can be assigned to the 5‐ and 7‐membered rings of the azulene unit.^15^ The peaks at 123 and 130 ppm were assigned to aromatic carbons of the phenyl units in the polymer backbone. The Fourier transform infrared spectroscopy (FTIR) spectrum of P‐Az‐B showed typical —C=C— stretching mode at ≈1570 cm^−1^ of the azulene unit (Figure 1d).^15^ The signals at around 1420 cm^−1^ indicate the skeleton vibration of the aromatic rings in the polymer, accompanied with the typical C—H stretching mode at 740 and 1090 cm^−1^. The UV–vis diffuse reflectance spectrum (DRS) revealed a broad absorption band of P‐Az‐B ranging from the visible into near‐infrared region (<1200 nm), as displayed in Figure 1e. An optical band gap of 2.03 eV could be derived from the Tauc plot (Figure 1f).

For photocatalytic systems, the energy band positions represent their photogenerated redox potentials and are therefore the crucial parameters for the catalytic efficiency. The highest occupied molecular orbital (HOMO) and the lowest unoccupied molecular orbital (LUMO) of P‐Az‐B were determined to be +1.14 and −1.10 V versus saturated calomel electrode (SCE) via cyclic voltammetry analysis (Figure 1f and Figure S3 (Supporting Information)). These values are comparable with the redox potentials of well‐established transition metal complex photocatalysts such as [Ru(bpy)_3_]^3+^ (+1.29 V vs SCE) and [Ru(bpy)_3_]^2+^ (−0.81 V vs SCE).^7^ This might indicate a possible catalytic capability of P‐Az‐B for photoredox reactions. Theoretical calculations using the density functional theory (DFT) at B3LYP/6‐31G(d) level showed an electron density separation between azulene and the phenyl moieties on the HOMO and LUMO for P‐AZ‐B (Figure S6, Supporting Information).

For the aryl radical‐mediated arylation reactions reported in the literature,^4^, ^5^ the aryl radicals were obtained via dehalogenation reaction of aryl halides, which was driven by the photogenerated electron of the photocatalyst. In contrast, for the here studied photocatalytic Stille‐type C—C bond formation reaction, we envision to report the formation of the aryl radical from the aryl stannane via oxidation driven by the photogenerated hole of the photocatalyst. We first examined the visible light‐promoted destannylation reaction of various aryl stannanes using P‐Az‐B as photocatalyst under the irradiation of a white light emetting diole (LED) lamp (1.2 W m^−2^). The results are listed in **Scheme** **1**. It could be clearly determined that the aryl stannanes (1c, 1g, and 1h) with oxidation potentials higher than the HOMO of P‐Az‐B (+1.14 V vs SCE) could not be destannylated (Figure S7, Supporting Information). Interestingly, the photo‐destannylation of the aryl stannanes with oxidation potentials lower than +1.14 V (vs SCE) led not only to the protonated aromates, but also to their dimers, except furanyl stannane (1a). It could be explained that thionyl (1b), indolyl (1d), benzothionyl (1e), and benzoethynyl (1f) could stabilize the radical aryl intermediate more efficiently and rather enhance the dimer formation (Figure S8, Supporting Information). The feasibility of the destannylation reaction of the aryl stannanes corresponded to the theoretical calculation of the free energy as listed in Table S5 (Supporting Information). To prove the formation of the aryl radical intermediate, we then conducted the destannylation reaction of benzoethynyl stannane in deuterated tetrahydrofuran (THF‐_d8_), leading to the corresponding deuterated aryl compound (Figure S9, Supporting Information). A radical trapping experiment via electron spin resonance spectroscopy was conducted using furanyl stannane as model substrate (Figure S10, Supporting Information). By adding *N*‐*tert*‐butyl‐α‐phenylnitrone as radical trapping agent,^16^ a characteristic signal pattern of the stabilized radical could be determined, demonstrating an aryl radical formation under the photocatalytic condition.

**Scheme 1:**
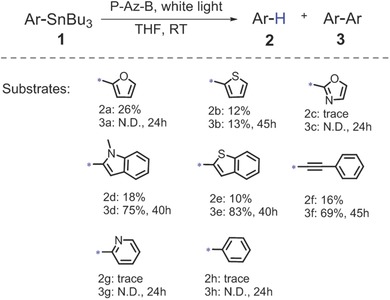
Photocatalytic destannylation reaction of various aryl stannanes using P‐Az‐B as photocatalyst. Reaction conditions: aryl tributylstannane (0.2 mmol), 5 mg P‐Az‐B (9 mol%), 5 mL THF, white LED (1.2 W cm^−2^), room temperature, air, 24–45 h. Yield determined via GCMS.

We then conducted the photoactivation of different aryl iodides as the second half reaction of the Stille coupling. We first exanimated the reductive ability of the photocatalyst whether P‐Az‐B was able to completely cleave the C—I bond of the aryl iodides into their dissociated states, i. e., an aryl radical and iodide anion. Significantly, none of the reactions led to the dehalogenated product (Table S6, Supporting Information). The reason was obviously that the photogenerated electrons from the LUMO level of P‐Az‐B (−1.10 V vs SCE) was not sufficient enough for the complete dissociation of the C—I bond of the aryl iodides. A clear indication could be derived from the determination of the reduction potential of 4‐iodobenzonitril via cyclic voltammetry (Figure S11, Supporting Information). Here, two reduction waves of 4‐iodobenzonitril could be observed. The first reduction onset potential *E*_red. 1_ was at −0.85 V versus SCE, and the second reduction onset potential *E*_red. 2_ was at −1.89 V versus SCE. Only the second reduction wave corresponded to the complete dissociated state of the aryl halide, which lay significantly higher than the HOMO of P‐Az‐B (−1.10 V vs SCE). The first reduction potential represented the so‐called activated state by forming an anionic radical of the aryl halide with no dissociation. This could explain the no observation of the dehalogenated products as demonstrated in Table S6 (Supporting Information). Further cyclic voltammetry measurements showed that most aryl iodides exhibited two reduction waves, as illustrated in Figure S12 (Supporting Information), which corresponded to the literature.^17^ Especially the aryl iodides containing electron‐withdrawing groups as such NO_2_, CN, carbonyl, or methyl benzoate showed a lower first reduction potential than the LUMO of P‐Az‐B, indicating a possible formation of their anionic radical by receiving one photogenerated electron. Xiao et al. showed via the DFT calculation, when an electron enters an unoccupied orbital of the iodobenzene molecule, the C—I bond elongates from 0.214 to 0.300 nm,^18^ which could enable the feasibility of the possible substitution reaction of the iodide.

Based on the obtained observation in both half cycles during the reaction, we propose a possible reaction mechanism for the photocatalytic Stille‐type C—C bond formation reaction, as illustrated in **Figure** **2**. The photocatalytic pathway is driven by the coupling reaction of the photogenerated aryl radical, which was obtained via oxidative destannylation by the photogenerated hole of the organic photocatalyst, with the activated radical anion of the aryl halide by the photogenerated electron. The reductive activation of the aryl halide by the photogenerated electron was mandatory for the successful coupling reaction. The attack of the activated radical anion of the aryl halide by the aryl radical led to a possible substitution mechanism, as the aforementioned elongation of the C—I bond by receiving one electron. The final C—C bond could be formed by releasing the iodide anion.

**Figure 2:**
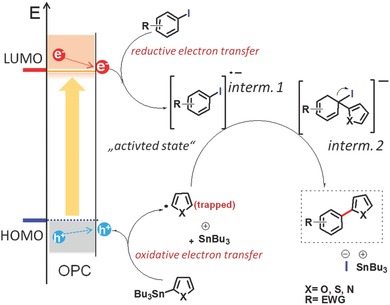
Proposed reaction mechanism of the photocatalytic Stille‐type coupling reaction using conjugated organic catalysts as photocatalyst. OPC: organic photocatalyst; interm.: intermediate; EWG: electron withdrawing group.

To study the general applicability of the photocatalytic Stille‐type reaction cycle, we then further examined the coupling reaction of various aryl iodides and stannanes. The substrates and products are listed in **Table** **1**. It could be observed that only the Stille‐type coupling of electron withdrawing group‐substituted aryl iodides with activated energy lower than the LUMO of P‐Az‐B (−1.10 V vs SCE) with aryl stannanes was successful (entries 1–12). Electron‐donating group or unsubstituted phenyl iodides did not lead to the formation of products (entries 13, 14). As expected, the employment of unreactive aryl stannanes with higher oxidation potentials, as shown in the aforementioned destannylation study, led to no desired coupling product (entries 15–17). A steric effect could be seen using 1‐iodo‐2‐nitrobenzene, which could not be coupled with the aryl stannane (entry 18). The reaction of aryl bromide and chloride with aryl stannane did not lead to any product. This could be explained by their high reduction potentials as shown in Figure S12 (Supporting Information). A potential solution to lower the reduction energy of the aryl bromides or chlorides (entries 19, 20) could be the introduction of more electron withdrawing substitutions on the aromatic ring. Additional experiments using alkyl substituted iodides or alkyl stannanes did not lead to the coupled products. This could be caused by the higher reduction potential of the alkyl iodides and oxidation potential of the alkyl stannanes compared to the corresponding aryl iodides or stannanes.

**Table 1:**
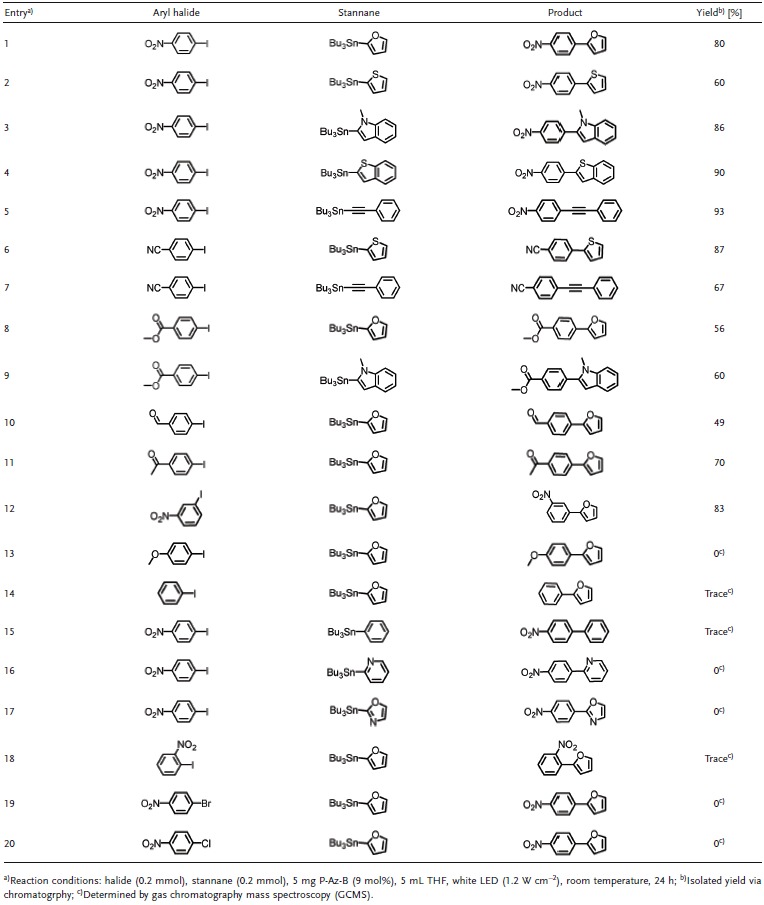
Scope of the photocatalytic Stille‐type coupling reaction with various substrates using P‐Az‐B as photocatalyst

The organic photocatalyt P‐Az‐B still contained a minimal palladium residue of ≈7 ppm according to inductively coupled plasma atomic emission spectroscopy. To eliminate the effect of the palladium residue during the Stille‐type coupling reaction, we synthesized a linear oligomer (L‐Az‐B) based on azulene and phenyl units, a soluble version to P‐Az‐B (Pd content <1 ppm). In a homogeneous manner, desired products were obtained in high yields (Table S7, Supporting Information).

Additional control experiments showed no product formation using P‐Az‐B as catalyst in dark. Using heat in dark led to no production formation either. These observations demonstrated that the photocatalytic activity was derived from the conjugated polymer backbone structure rather than the minimal Pd residue, which was proved by using soluble small molecule organic semiconductor‐based photocatalytic systems as reported.^4^, ^13^

It is worth to mention that P‐Az‐B could be used for five repeating cycles without significant change in its catalytic efficiency (Figure S13, Supporting Information). No clear change of the FTIR spectra and SEM images of P‐Az‐B was observed (Figures S14 and S15, Supporting Information). Using a single wavelength blue LED lamp (460 nm, 0.26 W cm^−2^), the apparent quantum yield of P‐Az‐B could be calculated as 0.09% (see the Supporting Information).

To further study the mechanistic insight of the photocatalytic Stille‐type coupling reaction, we then preformed time‐resolved photoluminescence spectroscopy using L‐Az‐B as photocatalyst and furanyl stannane and iodobenzene as substrates to mimic the catalytic system. As shown in **Figure** **3**, the photoluminescence exhibited a biphasic decay. While the fast component is in the order of 0.1 ns for all four measurements, the slower component of the decay varies with sample composition. The slow component of the photoluminescence L‐Az‐B has lifetime of 1.9 ns. By adding furanyl stananne, the lifetime was reduced to 1.5 ns. Adding iodobenzene decreases the lifetime of L‐Az‐B to 1 ns. By adding both coupling partners, the photoluminescence lifetime was reduced to 0.9 ns. This demonstrated a photoinduced electron transfer between the photocatalyst and the substrates during the catalytic cycle.

**Figure 3:**
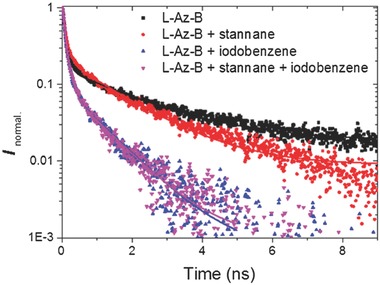
Fluorescence lifetime quenching experiments with L‐Az‐B by aryl stannane and iodide.

In summary, we demonstrated a novel photocatalytic Stille‐type coupling reaction pathway for aromatic C—C bond formations using conjugated organic photocatalysts instead of palladium complexes. The coupling reaction occurred between the aryl radical generated by oxidative destannylation of the aryl stannane, and the electron‐activated aryl iodide. The driven force was the photogenerated electron/hole pair of the conjugated organic photocatalysts. The visible light‐promoted Stille coupling reaction using pure organic photocatalysts offers a simple, sustainable, and more economic synthetic pathway toward metal‐free C—C bond formation, and can be applied for a broader range of coupling reactions. As a further perspective, we believe that nontoxic cleavable substituents other than stannanes, for example, pseudohalides, if the oxidation potential matches well with the photocatalysts, could also be chosen as a suitable coupling partner during the catalytic cycle. To extend the scope of substrates such as vinyl‐containing compounds, alkyl halides or stannannes, further design of the conjugated organic photocatalysts with more sufficient redox potentials is needed.

## Experimental Section

*Photocatalytic Destannylation Reaction of Aryl Stannanes Using P‐Az‐B as Photocatalyst*: A 25 mL Schlenk tube was filled with 5 mg P‐Az‐B, 70 µL (0.2 mmol) aryl stannane in 5 mL THF. The reaction mixture was kept under stirring in air while it was irradiated with a white LED lamp (1.2 W cm^−2^) for 24–45 h. After the reaction was finished, the catalyst was removed by filtration and the raw product was purified by column chromatogrphy with hexane/ethylacetate (5:1 volume ratio) as eluent.

*General Procedure for the Photocatalytic Stille‐Type Coupling of Aromatic Iodides with Stannanes*: A 25 mL Schlenk tube was filled under argon atmosphere with 5 mg of P‐Az‐B, 0.2 mmol of ArI, and 0.2 mmol of the corresponding tri‐*n*‐butylstannane. Additional 4 mL dry THF was added and the mixture was irradiated with a white LED lamp (1.2 W cm^−2^) while stirring for 24 h. The product purification procedure was similar to the destannylation reaction.

## Conflict of Interest

The authors declare no conflict of interest.

## Supporting information

Supplementary
