# Supplementary material for: A Conjugated Microporous Polymer for Palladium‐Free, Visible Light‐Promoted Photocatalytic Stille‐Type Coupling Reactions
Source: Adv Sci (Weinh). 2017 May 22;4(8):1700101. doi: 10.1002/advs.201700101 (PMC5566346; doi:10.1002/advs.201700101)
Supplement: Supplementary file 1 — Supplementary [file ADVS-4-na-s001.pdf]

Copyright WILEY-VCH Verlag GmbH & Co. KGaA, 69469 Weinheim, Germany, 2017.

Supporting Information

for *Adv. Sci.,* DOI: 10.1002/advs.201700101

A Conjugated Microporous Polymer for Palladium-Free, Visible Light-Promoted Photocatalytic Stille-Type Coupling Reactions

*Saman Ghasimi, Simon A. Bretschneider, Wei Huang, Katharina Landfester, and Kai A. I. Zhang**

Copyright WILEY-VCH Verlag GmbH & Co. KGaA, 69469 Weinheim, Germany, 2013.

Supporting Information

A conjugated microporous polymer for palladium-free, visible light-promoted photocatalytic Stille-type coupling reactions

*Saman Ghasimi, Simon A. Bretschneider, Wei Huang, Katharina Landfester, and Kai A. I. Zhang**
Max Planck Institute for Polymer Research, Ackermannweg 10, 55128 Mainz, Germany

Materials and methods

All chemicals were purchased from commercial sources, unless otherwise specified. Solvents were in HPLC quality or extra dry. Water was used in Millipore quality.

The ^{1}H and ^{13}C NMR spectra were measured with Bruker Avance 300. The following resonance frequencies ^{1}H (300 MHz) and ^{13}C (75 MHz) were used. Solid state ^{13}C CP MAS NMR measurement was conducted on a Bruker Avance 300 NMR spectrometer with cross polarization and magic angle spinning technique at rotor speed of 10 kHz. Solid State UV-vis remission (DRS) measurements were taken from a Perkin Elmer Lambda 900 UV-vis/NIR spectrometer. UV-vis measurements in liquid phase were taken from Perkin Elmer Lambda 25 UV-vis spectrometer with Suprasil QS 1 mm cuvettes. The GC/MS measurement was conducted with Shimadzu QP 2100 plus in dichloromethane. Gel permeation chromatography (GPC) was performed on a PSS SecCurity (Agilent Technologies 1260 Infinity) with polystyrene as a molecular weight reference. The cyclic voltammograms were taken on the Metrohm Autolab PGSTAT204 potentiostat/galvanostat using tetrabutylammonium hexafluorophosphate (0.1 M) as electrolyte in dichloromethane. The measurments were carried out in a three electrode arrangement with glassy carbon as working electrode, platinum wire as counter electrode, and with the standard calomel electrode (SCE) as reference. The TGA measurements were conducted under nitrogen with increasing temperature from 25 ^{o}C to 1000 ^{o}C at a rate of 10 ^{o}C/min. Scanning electron microscopy (SEM) images were recorded with a low voltage high resolution scanning electron microscope (high vacuum) LEO Gemini 1530 with a wolfram emitter. Transmission electron microscopy images (TEM) were recorded with LaB_6 emitter at an acceleration voltage of 120kV with JEOL JEM 1400. Surface areas and pore size distributions of the porous polymers were measured by nitrogen adsorption and desorption at 77.3 K using Autosorb 1

(Quantachrome Instruments). The polymers were degassed at 100 °C for 24 h under vacuum before analysis. Data was obtained using QuadraWin software from Quantachrome Instruments. Pore size distributions and pore volumes were calculated from the adsorption branches of the isotherms using Quenched Solid Density Functional Theory (QSDFT, N2, evaluating carbon adsorbent with slit pores). The BET surface area was obtained based on data points received from $0<P/P0<0.25$ and the non-local density functional theory (NLDFT) equilibrium model was employed as the BET model fitting. The quantum mechanical calculations were performed with the Gaussian 09 program suite on a cluster system. The thermodynamic data of 4-iodonitrobenzene were obtained by applying a vibrational calculation on pre-optimized geometries on the semi-empirical level with the PM6 method at 1 atm and 298.15 Kelvin. The HOMO/LUMO molecular orbitals were calculated by applying density functional theory level with the Becke, three-parameter, Lee-Yang-Parr B3LYP hybrid functional and a 6-31G(d) split valence basis set. Inductively coupled plasma atomic emission spectroscopy (ICP-AES) was conducted on a Jobin-Yvon Activa M spectrometer.

Time-resolved photoluminescence (TRPL) on a nanosecond timescale was taken with a Streak Camera System (Hamamatsu C4742) in slow sweep mode. The excitation wavelength of 400 nm was provided using the frequency-doubled output of a commercial titanium:sapphire amplifier (Coherent LIBRA-HE, 3.5 mJ, 1 kHz, 100 fs). The data was fitted with a biexponential decay function.

Monomer synthesis

Bromination of azulene

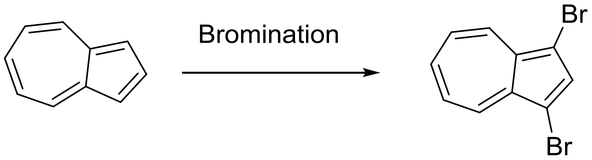

According to the literature[1] 500 mg (3.9 mmol, 1 eq.) azulene was dissolved in 50 ml THF and cooled to 0 °C. 1.53 g (8.6 mmol, 2.2 eq.) of N-bromosuccinimide were dissolved in 30 ml THF and cooled. The cold NBS solution was added dropwise under exclusion of light over a time period of one hour. The mixture was allowed to warm up to room temperature and stirred for 24 hours. The crude mixture was extracted by dichlormethane and washed several times with water. The combined organic phases were dried and concentrated. The final purification was achieved by column chromatography (SiO, hexane). 963 mg (3.4 mmol, 87%) of dark green crystalline material was obtained.

1H NMR 8.32 (d, 2H), 7.81 (s, 1H), 7.68 (m, 1H), 7.28 (t, 2H) ppm.

13C-NMR: (300 MHz, CDCl3, 25 °C): δ = 140, 138.2, 136.7, 135.7, 124 ppm.

Synthesis of 1,3-bis(phenyl)azulene (Az-Ph$_2$)

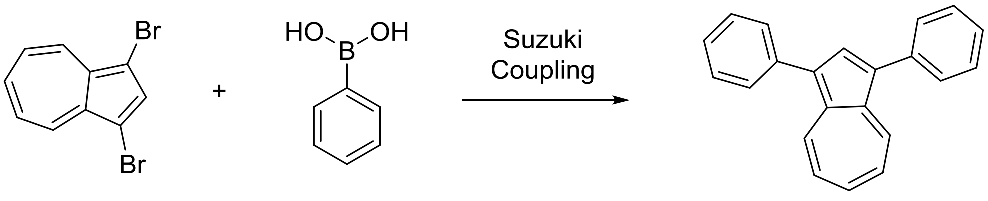

Az-Ph$_2$

A 25 ml Schlenk tube was filled under argon with 100 mg 1,3-dibromoazulene (0.35 mmol, 1 eq), 86 mg phenylboronic acid (0.70 mmol, 2 eq), 25 mg Pd(PPh$_3$)$_4$ (6 mol%). After dissolving the mixture in 4 ml THF, 1.5 ml of aqueous potassium carbonate (341 mg, 2.47 mmol, 7 eq) was added. The reaction mixture was left at reflux temperature while it was stirring about 24 h. After cooling down to room temperature, the reaction mixture was washed with brine and water, and extracted three times with 100 ml of dichloromethane. The organic phase was filtered over Celite® to remove the catalyst residue. After drying over $MgSO_4$. the solvent was removed under reduced pressure. The crude product was purified

with column chromatography with hexane as eluent. 36 mg (37%) of dark blue solid was obtained.

^{1}H NMR (300 MHz, CDCl3, 25 °C): δ (ppm) = 8.45 (d, 2H), 8.04 (s, 1H), 7.55 (m, 4H), 7.50 (t, 1H), 7.43 (m, 4H), 7.28 (m, 2H), 7.03 (t, 2H)

Polymer synthesis

Synthesis of P-Az-B

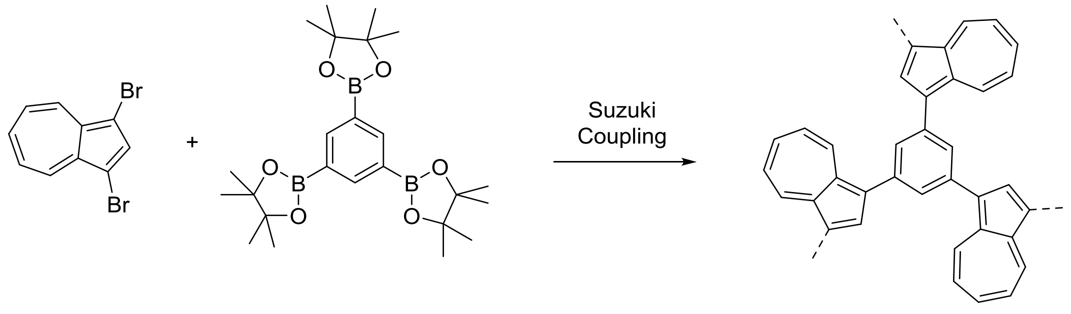

P-Az-B

A 25 ml Schlenk tube was filled under argon with 210 mg phenylboronic acid (0.46 mmol, 1 eq), 67 mg 1,3-dibromoazulene (0.23 mmol, 0.5 eq), 32 mg $Pd(PPh_3)_4$ (6 mol%). After dissolving the mixture in 6 ml DMF, 2 ml of aqueous potassium carbonate (4.45 mg, 3.22 mmol, 7 eq) was added. The reaction mixture was heated at 90 °C under continuous stirring. After 24 hours additional 67 mg 1,3-dibromoazulene (0.23 mmol, 0.5 eq) was added to the reaction mixture. After another 24 hours, the last step was repeated and again 67 mg 1,3-dibromoazulene (0.23 mmol, 0.5 eq) was added to the reaction mixture, while keeping the reaction for 24 hours at 90°C. Finally the reaction mixture was refluxed at 145 °C for further 24 hours. Each addition step was accompanied with a change of color from pale green, dark blue, to black. After cooling down to room temperature, the reaction mixture was filtered, washed several times with brine, water, and several times with dichloromethane and THF. The black powder was extracted by Soxhlet extraction (MeOH, DCM) for 2 days. Yield: 108 mg (52 %).

Synthesis of L-Az-B

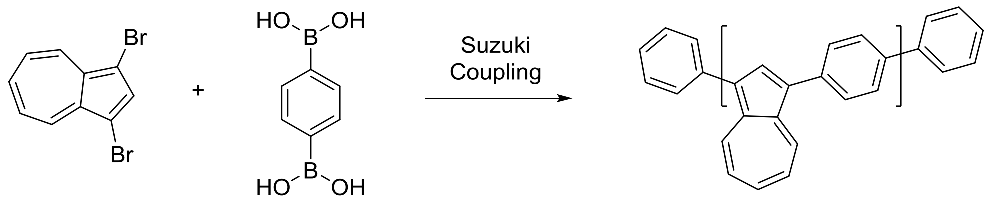

A 25 ml Schlenk tube was filled under argon with 142 mg 1,3-dibromoazulene (0.5 mmol, 1 eq), 83 mg phenylboronic acid (0.5 mmol, 1 eq), 35 mg $Pd(PPh_3)_4$ (6 mol%). After dissolving the mixture in 4 ml THF, 2.3 ml of aqueous potassium carbonate (484 mg, 3.5 mmol, 7 eq) was added. The reaction mixture was left at reflux temperature while it was stirring about 48 h. Additional 61 mg phenylboronic acid (0.5 mmol) were added as endcapping agent. The mixture was again refluxed for 6 h before cooling down to room temperature. The reaction mixture was washed with brine and water, and extracted three times with 100 ml of dichloromethane. The organic phase was filtered over cellite to remove traces of precipitations of the catalysts. After drying over $MgSO_4$ the solvent was removed under reduced pressure. The crude product was purified with column chromatography. The final product was precipitated in cold methanol. 83 mg (82%) of dark green powder was obtained.
M_w = 1023 g/mol, PDI = 1.27, ICP: Pd content: 0.01 ppm

Photocatalytic destannylation of tributyl(phenylethynyl)tin in deuterated THF

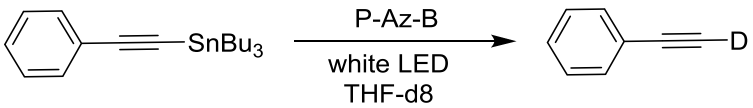

A 25 ml Schlenk tube was filled with 5 mg P-Az-B, 70 µl (0.2 mmol) tributyl(phenylethynyl)tin and 5 ml THF-$_{8d}$. The reaction mixture was kept under stirring in air while it was irradiated with a white LED lamp (1.2 W/cm^2, OSA Opto Lights) for 24 hours. After the reaction was finished the catalyst was removed by filtration and the raw product was purified by column chromatography with hexane/ethylacetate (5:1 volume ratio) as eluent.

Radical trapping experiment with N-tert-butyl-α-phenylnitrone (PBN)

A 20 ml vial was filled under argon atmosphere with 5 mg P-Az-B, 65 µl 2-(tributylstannyl)furan, 0.05M PBN, and 4 ml ahydrous THF. The mixture was irradiated under stirring with a white LED lamp. Samples were taken at different times and measured by EPR. In order to exclude other interactions of radical species with PBN, a control experiment was conducted with P-Az-B and PBN in THF.

Repeating experiments of the photocatalytic Still-type coupling of 4-iodonitrobenzene with 2-(tributylstannyl)furan

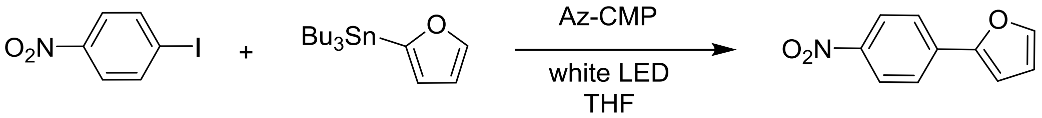

A 25 ml Schlenk tube was filled under argon atmosphere with 20 mg of P-Az-B, 0.2 mmol of 4-iodonitrobenzene, and 0.2 mmol of 2-(tributylstannyl)furan. Additional 4 ml dry THF was added and the mixture was irradiated with a white LED lamp (1.2 W/cm², OSA Opto Lights) while it was stirring for 24 hours. After the reaction was finished the catalyst was recovered by membrane filtration and the raw product was purified by column chromatography with hexane/ethylacetate (5:1 volume ratio) as eluent. The same batch of P-Az-B was reused in 5 consecutive runs by the above mentioned procedure.

Table S1: Porosity data of P-Az-Bs obtained in different solvents and in comparison with the stepwise addition method of the monomer.

Solvent	S_{BET} (m^2/g)	Pore Size (nm)	Pore Volume (cm^3/g)
THF	6	3.3	0.009
Dioxane	8	2.9	0.014
DMF	17	1.5	0.024
DMF (successive addition of monomers)	292	1.5	0.28

Table S2. Optical properties of P-Az-B and L-Az-B.

**Polymer**	**UV/vis λ_{max} (nm)**	**Optical band gap (eV)**
**P-Az-B**	590	2.03
**L-Az-B**	410 (strong), 490 (weak)	2.38

Table S3. Electrochemical properties of P-Az-B and L-Az-B.

	**HOMO [V vs. SCE]**	**LUMO [V vs. SCE]**	**Band gap [eV]**
**P-Az-B**	1.14	-1.10	2.32
**L-Az-B**	1.52	-1.08	2.60

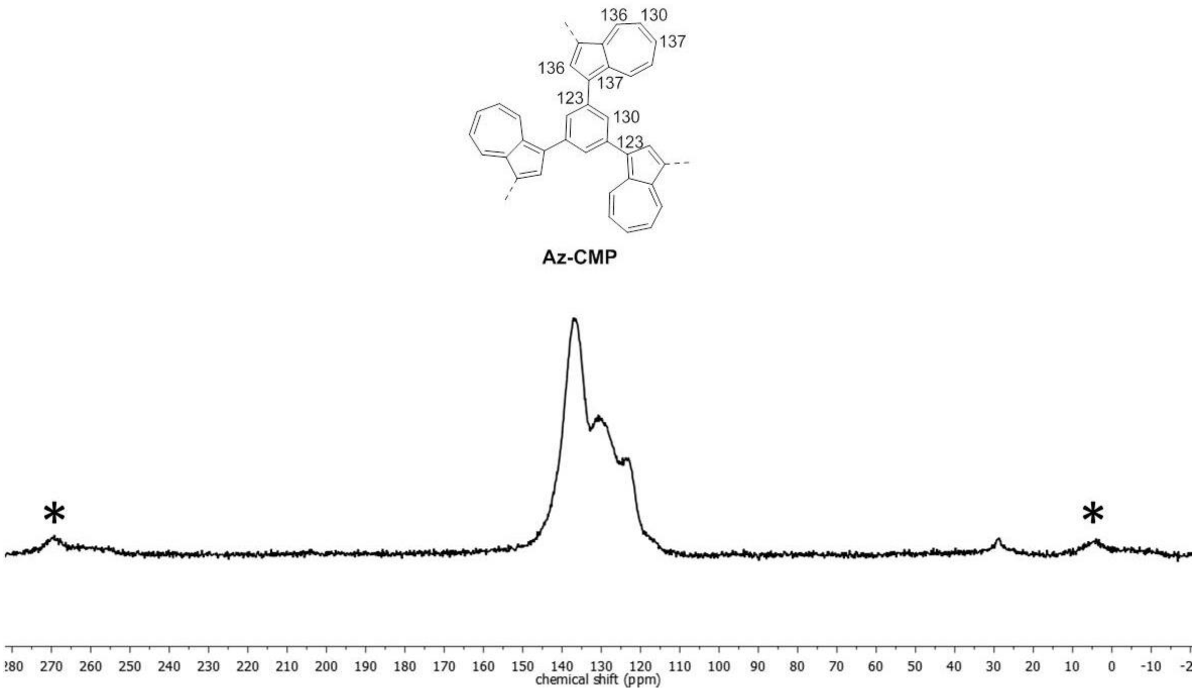

Figure S1: Solid state CP-MAS ^{13}C-NMR spectrum of P-Az-B at rotation frequency of 10 kHz (side bands*).

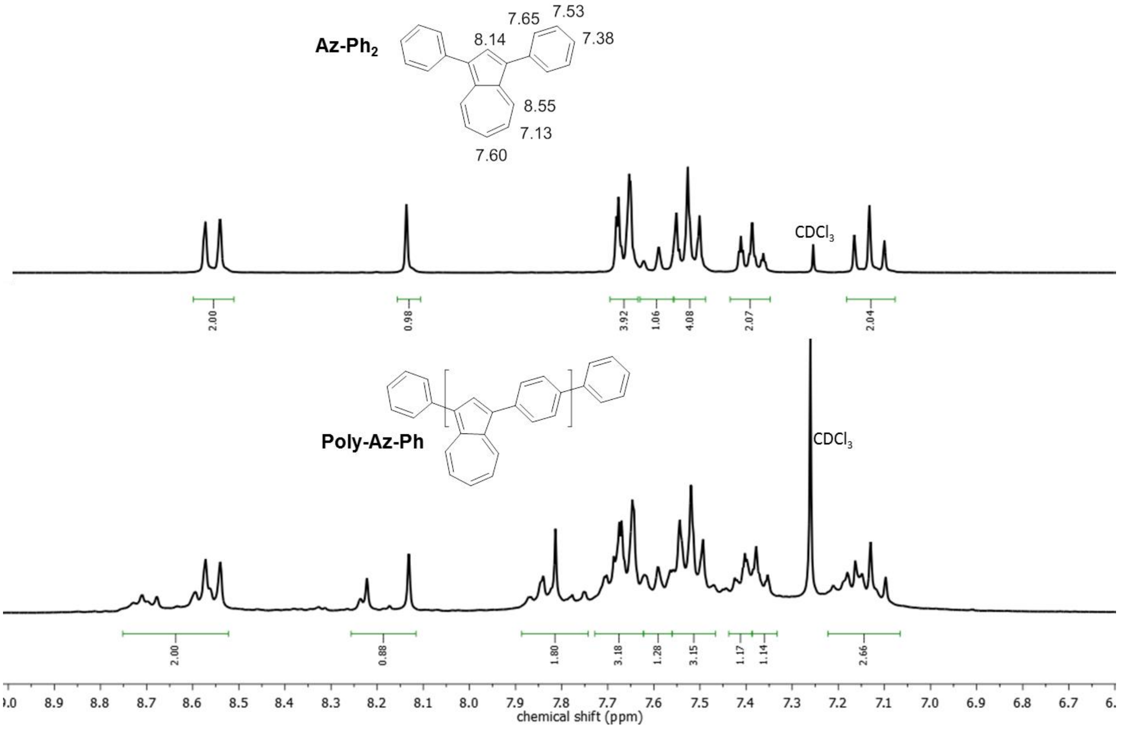

Figure S2: ^{1}H-NMR spectrum of Az-Ph_2 and L-Az-B.

Optimization of synthetic routes for P-Az-B

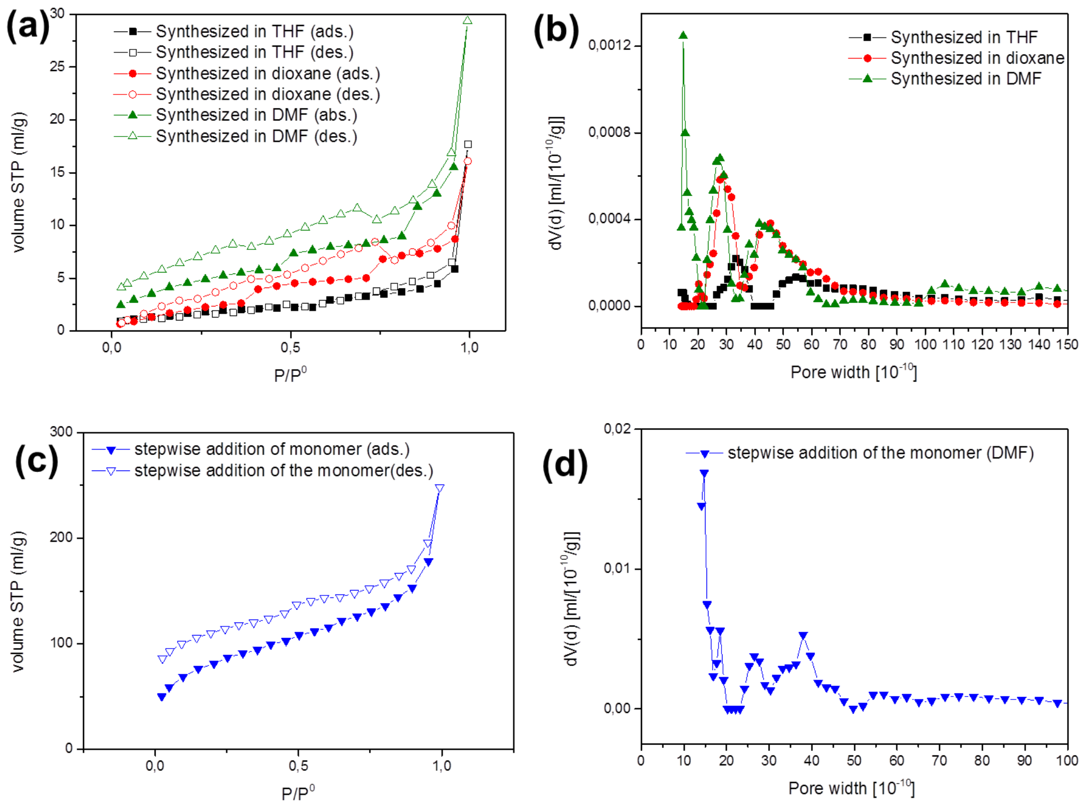

Figure S3: Gas sorption isotherms of the P-Az-B synthesized in (a) different solvents, (b) via stepwise addition of monomers during the polymerization, and (c) pore size distributions of P-Az-B synthesized in different solvents, and (d) via stepwise addition of monomers.

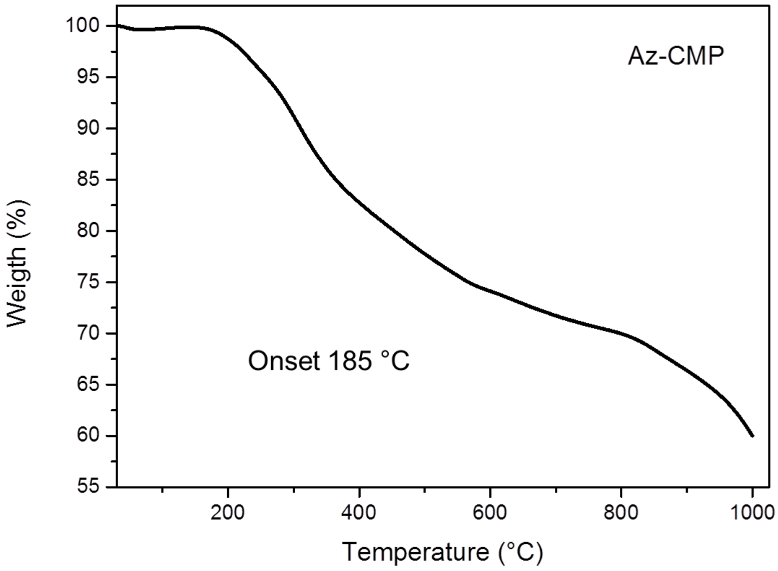

Figure S4: Thermogravimetric analysis of P-Az-B from 25 to 1000°C under nitrogen.

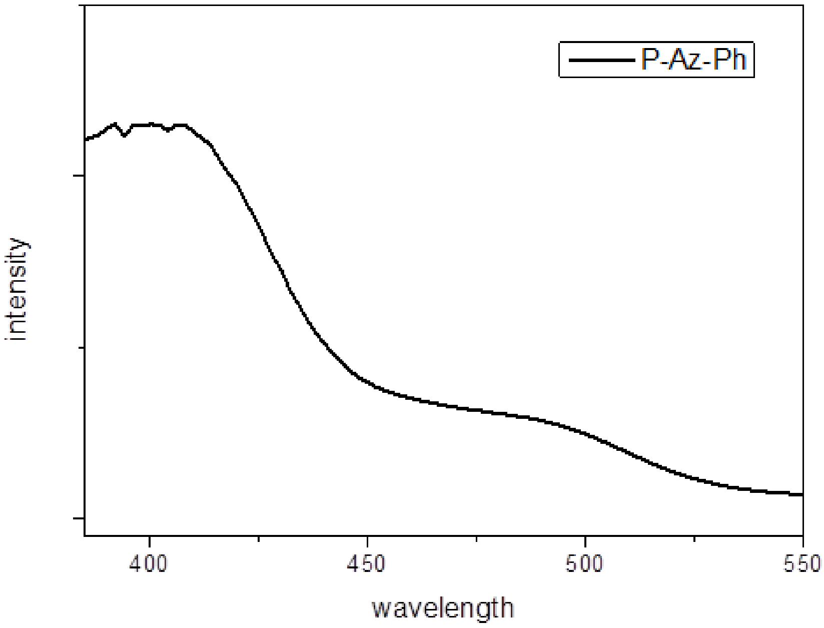

Figure S5: UV/vis absorption and emission spectra of the linear oligomer L-Az-B.

Table S4: Molecular Orbital levels and the corresponding bandgaps of pre-optimized structures (DFT, RB3LYP, 631G(d)) [2] of fragments of P-Az-B.

	**HOMO (V vs SCE)**	**LUMO (V vs SCE)**	**Bandgap (eV)**
**$PhAz_3$**	+0.76	-2.29	3.05
**Ph_4Az_9**	+0.62	-2.27	2.89

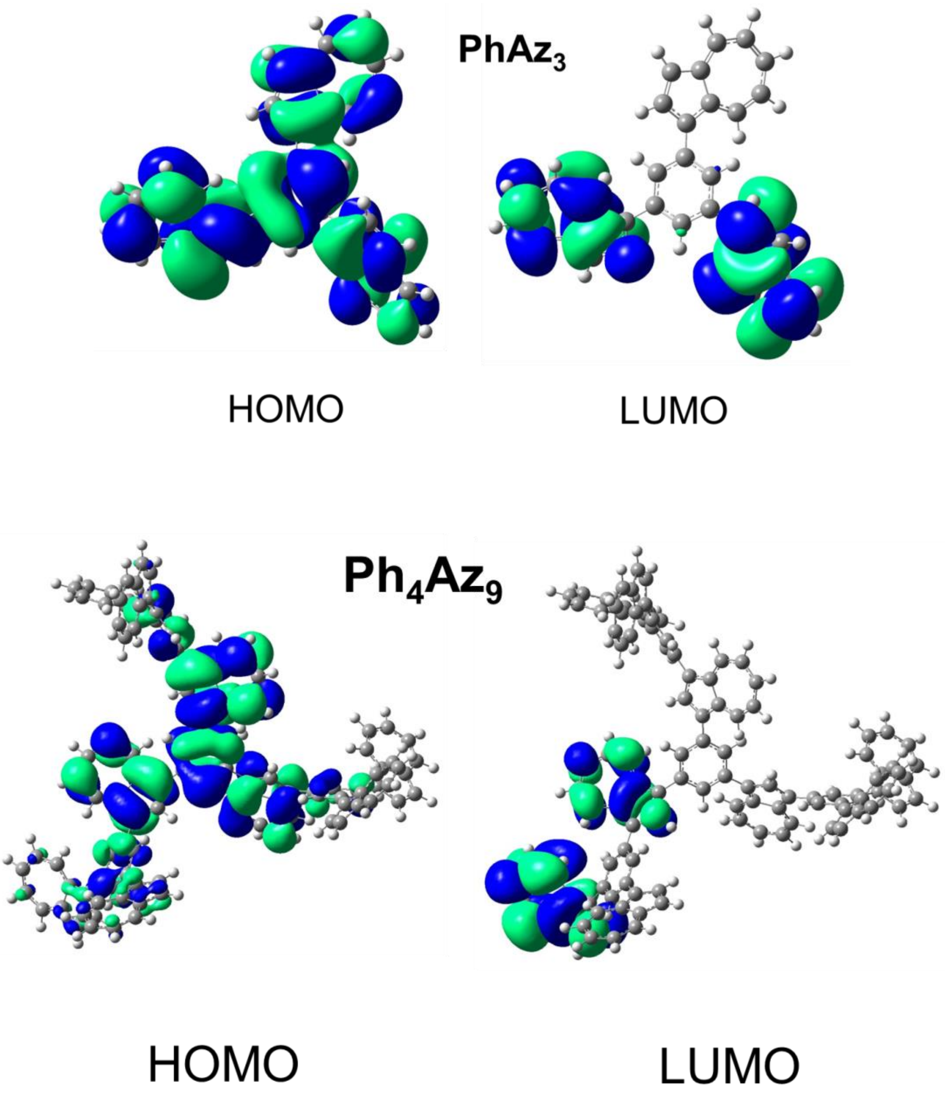

Figure S6: Molecular Orbital distribution of repeating fragments of P-Az-B (DFT, RB3LYP, 631G(d)).

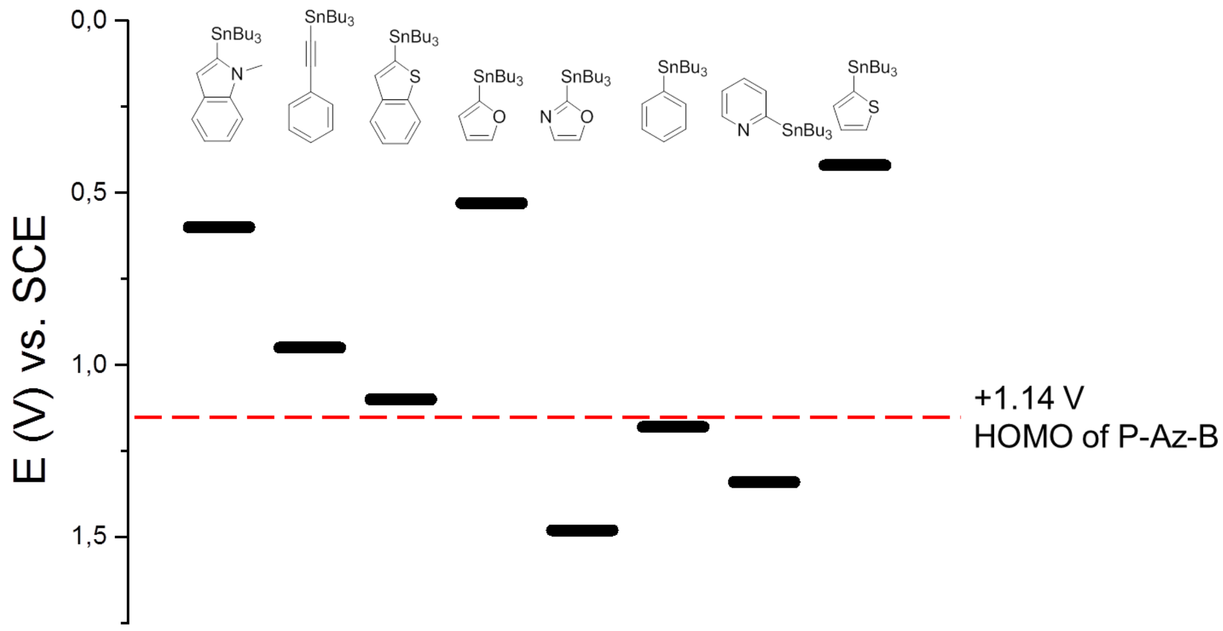

Figure S7: Oxidation potentials of the aryl stannanes compared to the HOMO level of P-Az-B, determined by cyclic voltammetry (vs. SCE).

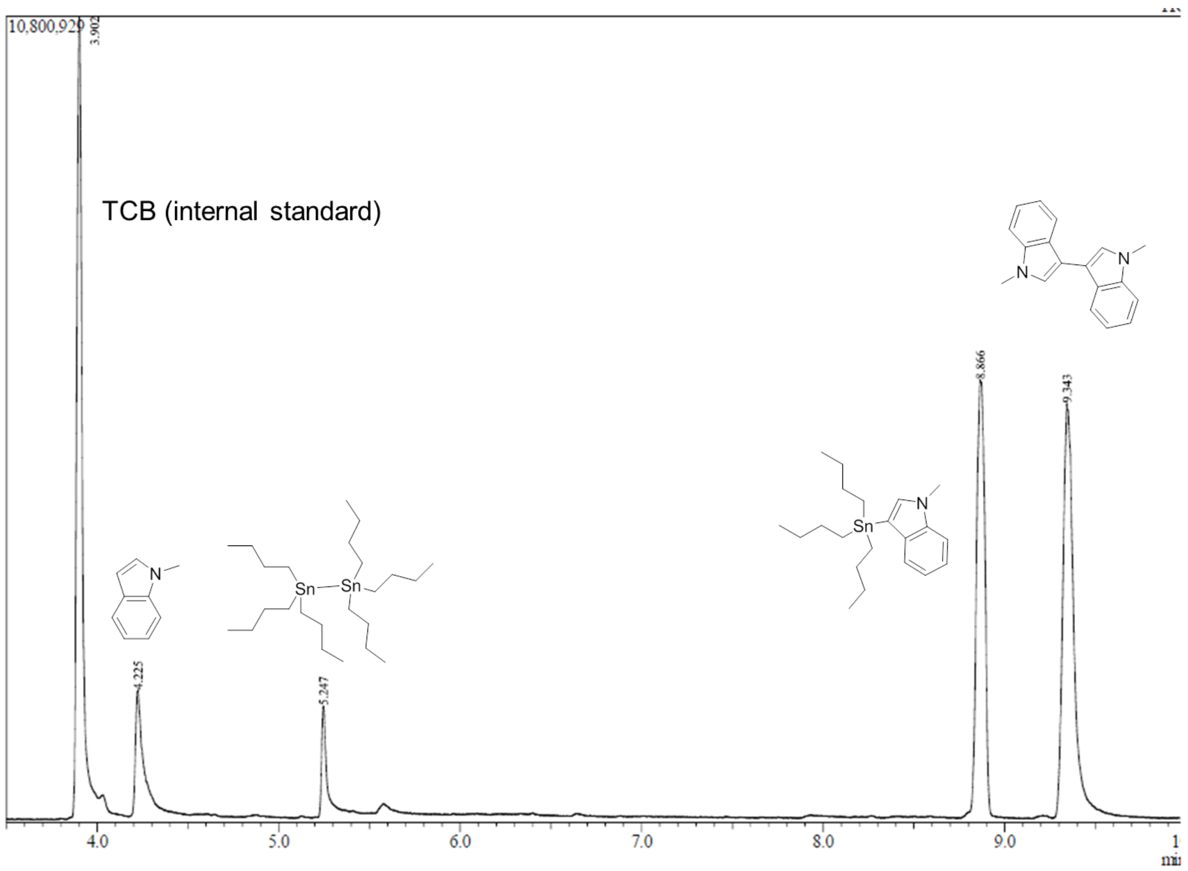

Figure S8: Photooxidation analysis of N-methyl-2-(tributylstannyl)indole by GC-MS after 40 h light irradiation.

Table S5: Calculated free energies of photo-induced electron transfer processes from the aryl stannanes to P-Az-B according to the Gibbs equation: $\Delta G = E_{ox}(D/D^{+}) - E_{red}(A/A^{-}) - \Delta E_{00} + \Delta E_{Coloumb.}$

	**$E_{red}(A/A^{-})$ (kcal/mol)**	**E_{0-0} (kcal/mol)**	**Stannanes**	**$E_{ox}(D/D^{+})$ (kcal/mol)**	**Free Energy (kcal/mol)**
P-Az-B	-35.1	69	1-methyl-2-(tributylstannyl)-1H-indole	14	-20
			tributyl(phenylethynyl)stannane	22	-12
			benzo[b]thiophen-2-yltributylstannane	25	-9
			tributyl(furan-2-yl)stannane	12	-22
			2-(tributylstannyl)oxazole	34	0
			tributyl(phenyl)stannane	27	-7
			2-(tributylstannyl)pyridine	31	-3
			tributyl(thiophen-2-yl)stannane	10	-24

D: donor species; A: acceptor species.

$E_{ox}(D/D^{+})$: oxidation energy of the donor

$E_{red}(A/A^{-})$: reduction energy of the acceptor

ΔE_{00}: excited singlet state energy

$\Delta E_{Coloumb}$: Coulombic potential energy

Negative values of the Gibbs energy indicate favored processes.[3]

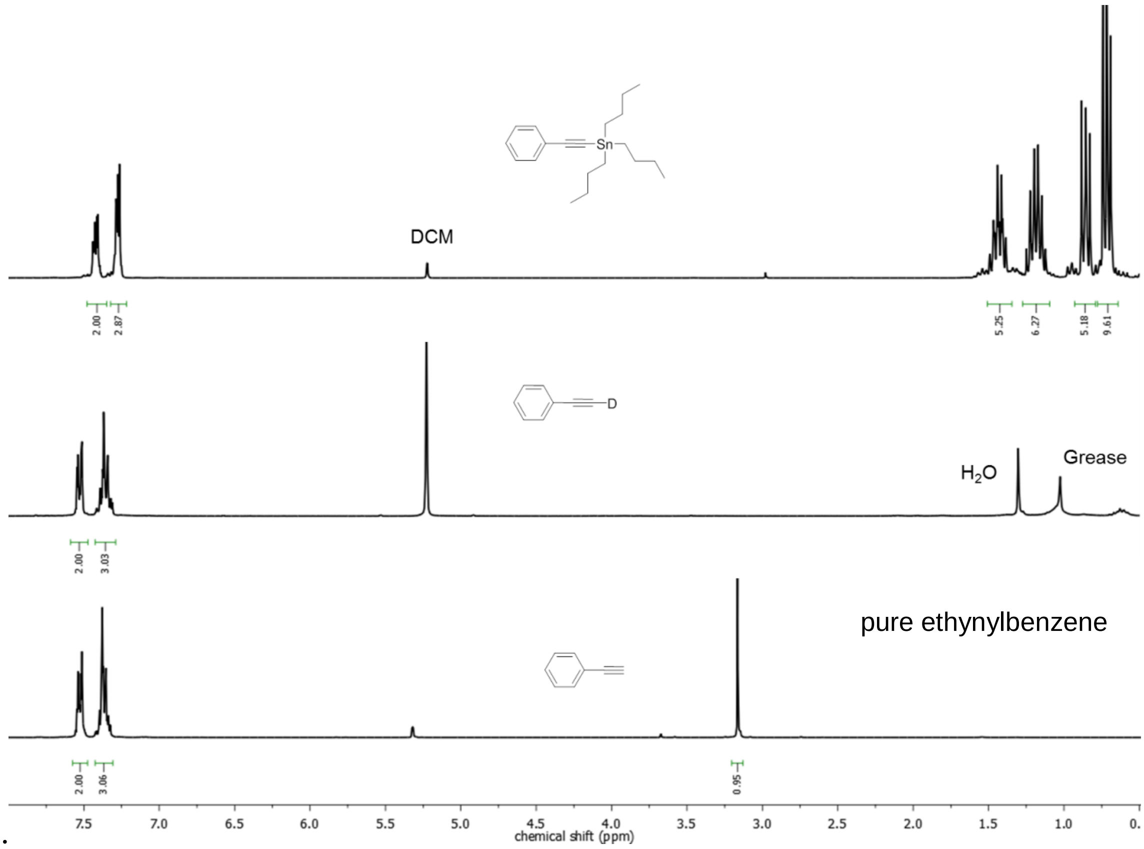

.

Figure S9: Photooxidation of tributyl(phenylethynyl)tin in deuterated THF compared to the substrate and pure ethynylbenzene.

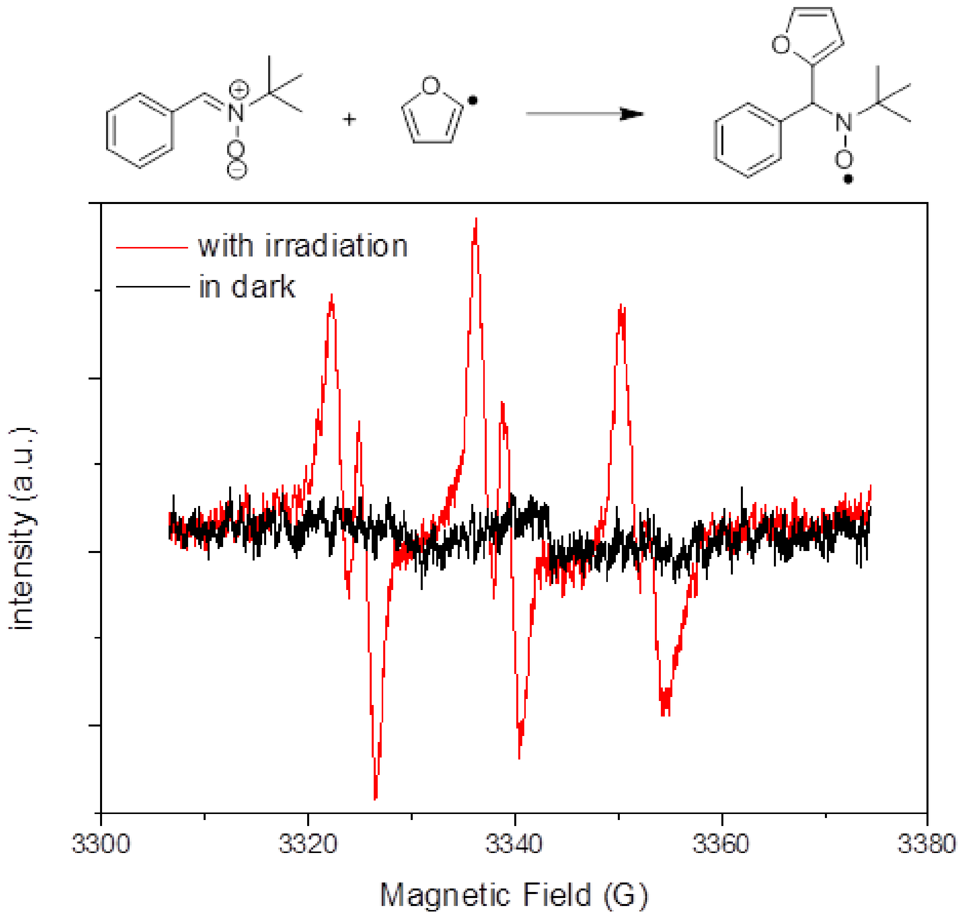

Figure S10: Photooxidation of 2-(tributylstannyl)furan in and trapping of the resulting radical by N-tert-Butyl-α-phenylnitrone (PBN).

Table S6. Dehalogenantion of aryl iodides using P-Az-B as photocatalyst

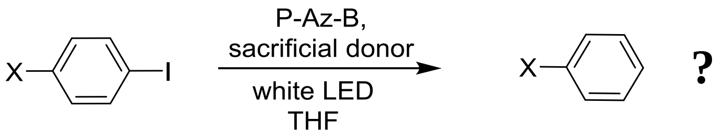

A 25 ml Schlenk tube was filled under argon atmosphere with 5 mg P-Az-B, 50 mg (0.2 mmol) aryl iodide, sacrificial donor and 4 ml dried THF. The mixture was irradiated with a white LED lamp (1.2 W/cm², OSA Opto Lights) for 24 hours. After the reaction was finished the catalyst was removed by filtration and the raw product was analyzed by GC/MS. It was found that no dehalogenated product could be found for all experiments (see Table below).

Substituent X	Sacrificial donor	amount (mmol)	Conversion of dehalogenation (%)
NO_2	triethylamine	0.4	0
NO_2	Hantzsch ester/triethylamine	0.2 / 0.4	0
NO_2	diisopropylamine	0.4	0
CN	triethylamine	0.4	0
Carbonyl	triethylamine	0.4	0
Methoxy	triethylamine	0.4	0
H	triethylamine	0.4	0

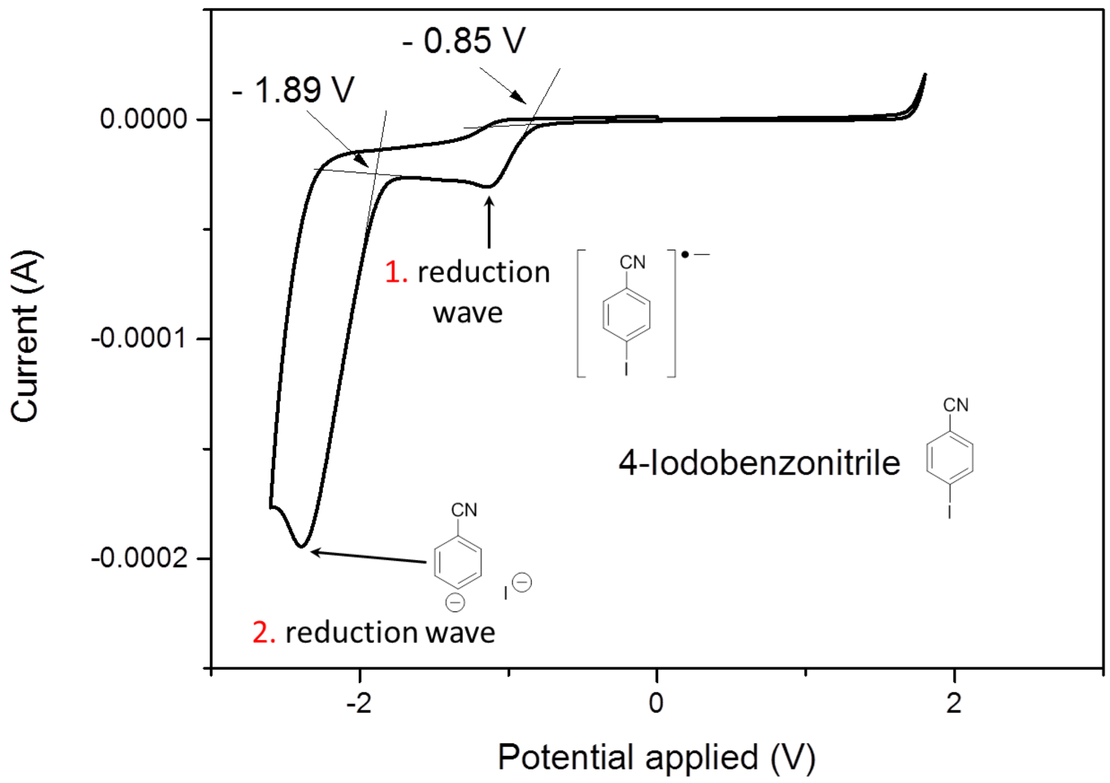

Figure S11: Cyclic voltammogram of 4-iodobenzonitril and the involved two electron reduction processe.

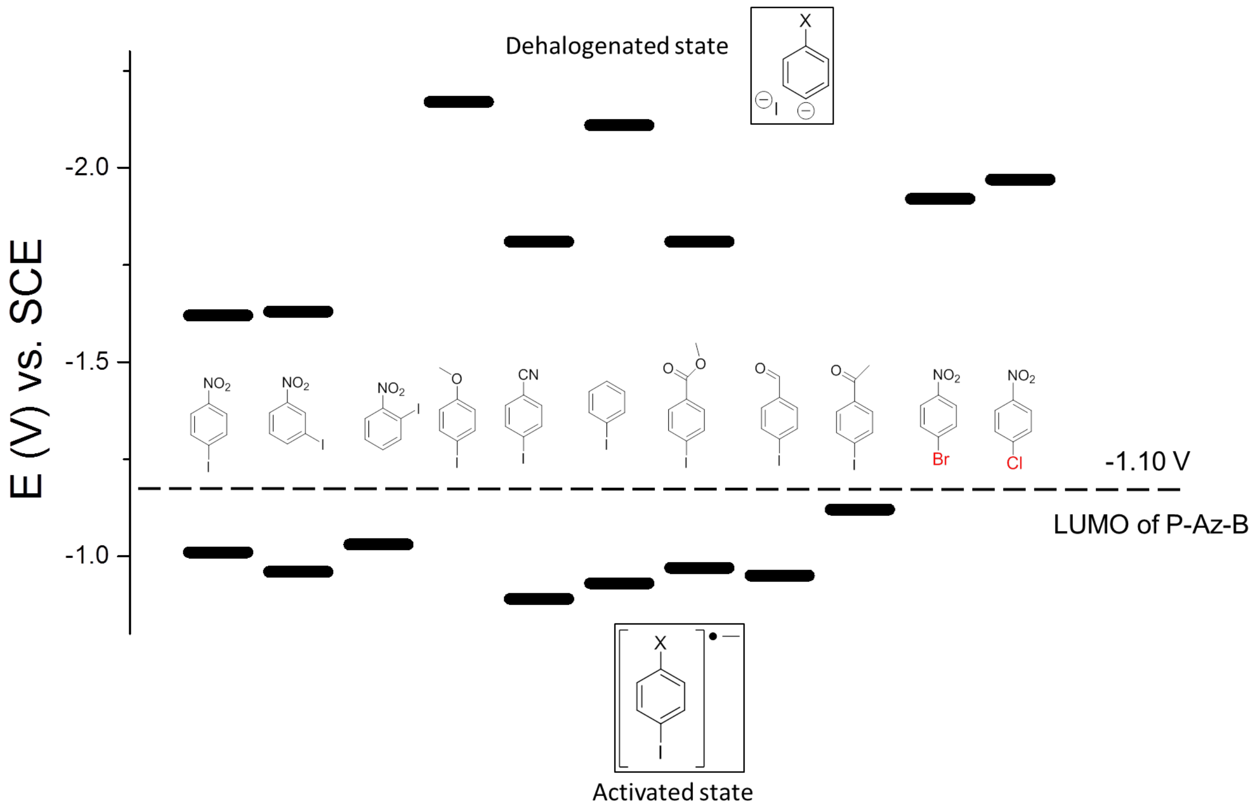

Figure S12: Reduction potential of aromatic halides compared to the LUMO of P-Az-B, determined by cyclic voltammetry (vs. SCE).

Table S7. Photocatalytic Stille-type coupling using the soluble oligomer L-Az-B as photocatalyst[a]

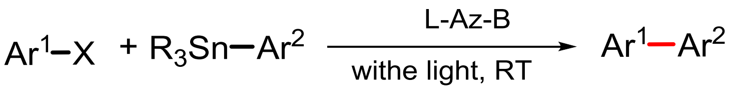

entry	aryl iodide	aryl stannane	product	yield (%)[b]
1	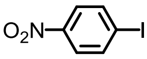	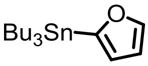	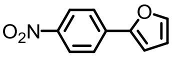	80
2	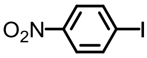	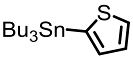	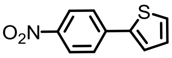	78
3	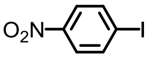	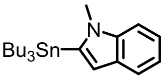	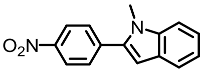	85
4	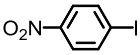	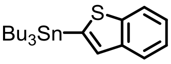	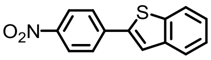	91
5	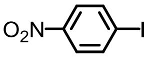	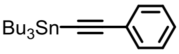	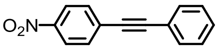	92

[a]Reaction conditions: halide (0.2 mmol), stannane (0.2 mmol), 5 mg L-Az-B, 5 ml THF, white LED (1.2 W/cm^2), room temperature, 24 h. [b]Isolated yield via chromatography.

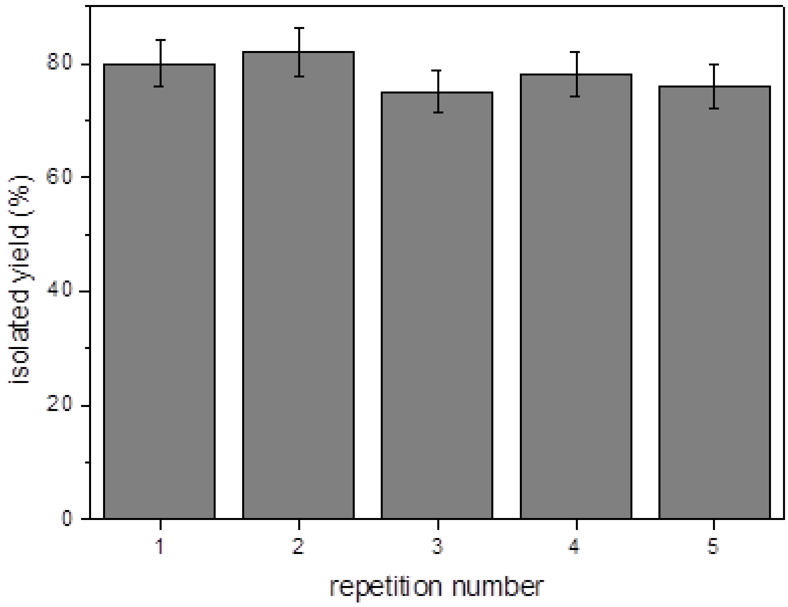

Figure S13: Repeating experiment of the photocatalytic Stille coupling of 4-iodonitrobenzene with 2-tri-n-butylstannylfuran by filtration and reusing the same P-Az-B photocatalyst after each repetition (isolated yields).

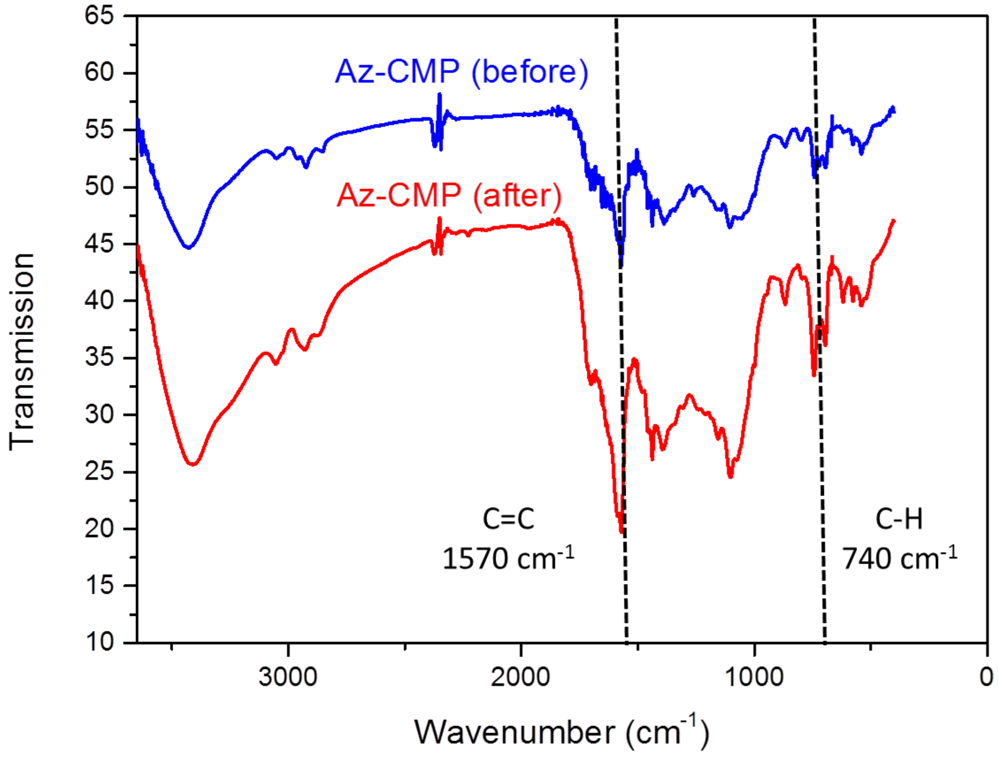

Figure S14: FT-IR spectra of P-Az-B before and after 5 cycles of photocatalysis.

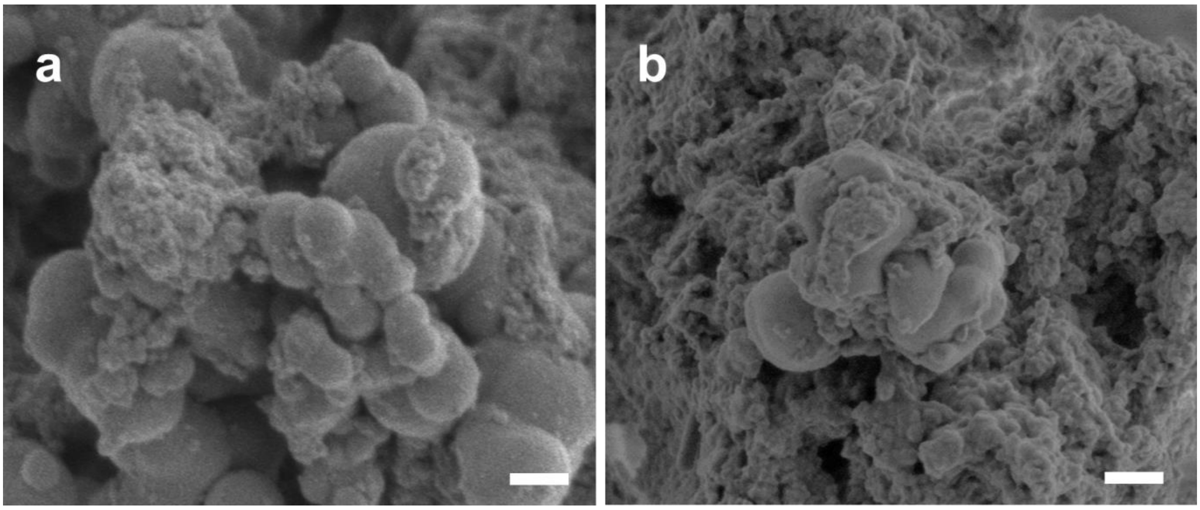

Figure S15: SEM image of porous P-Az-B before (a) photocatalytic cycles and after 5 subsequent cycles (b) (scale bar 200 nm).

Apparent Quantum Yield Measurements

In order to determine the apparent quantum yield, the photocatalytic Stille coupling was conducted by irradiating 5 mg P-Az-B, 50 mg (0.2 mmol) 4-iodonitrobenzen, and 63µl 2-(tributylstannyl)furan with a blue LED (460 nm, 0.26 W/cm^2). The conversion was determined after 1 hour by column chromatography. The illumination area was 6.76 cm^2 and the LED intensity was measured by a Coherent Lab-Max energy meter. The apparent quantum yield was estimated by following equation:

$$\Phi(AQY) = \frac{moles\ of\ product\ evolved}{moles\ of\ incident\ photons}$$

$$= \frac{N_e}{N_p} \times 100\% = \frac{M \times N_A}{\frac{E_{total}}{E_{photon}}} \times 100\%$$

$$= \frac{M \times N_A}{\frac{S \times P \times t}{\hbar \times \frac{c}{\lambda}}} \times 100\% = \frac{M \times N_A \times \hbar \times c}{S \times P \times t \times \lambda} \times 100\%$$

$$= 0.09\%$$

M is the amount of product (mol), N_A is Avogadro constant (6.022×10^{23}/mol), h is the Planck constant (6.626×10^{-34}J·s), c is the speed of light (3×10^8m/s), S is the irradiation area (cm^2), P is the intensity of irradiation light (W/cm^2), t is the photoreaction time (s), λ is the wavelength of the monochromatic light (m).

1H and 13C NMR spectra of the products

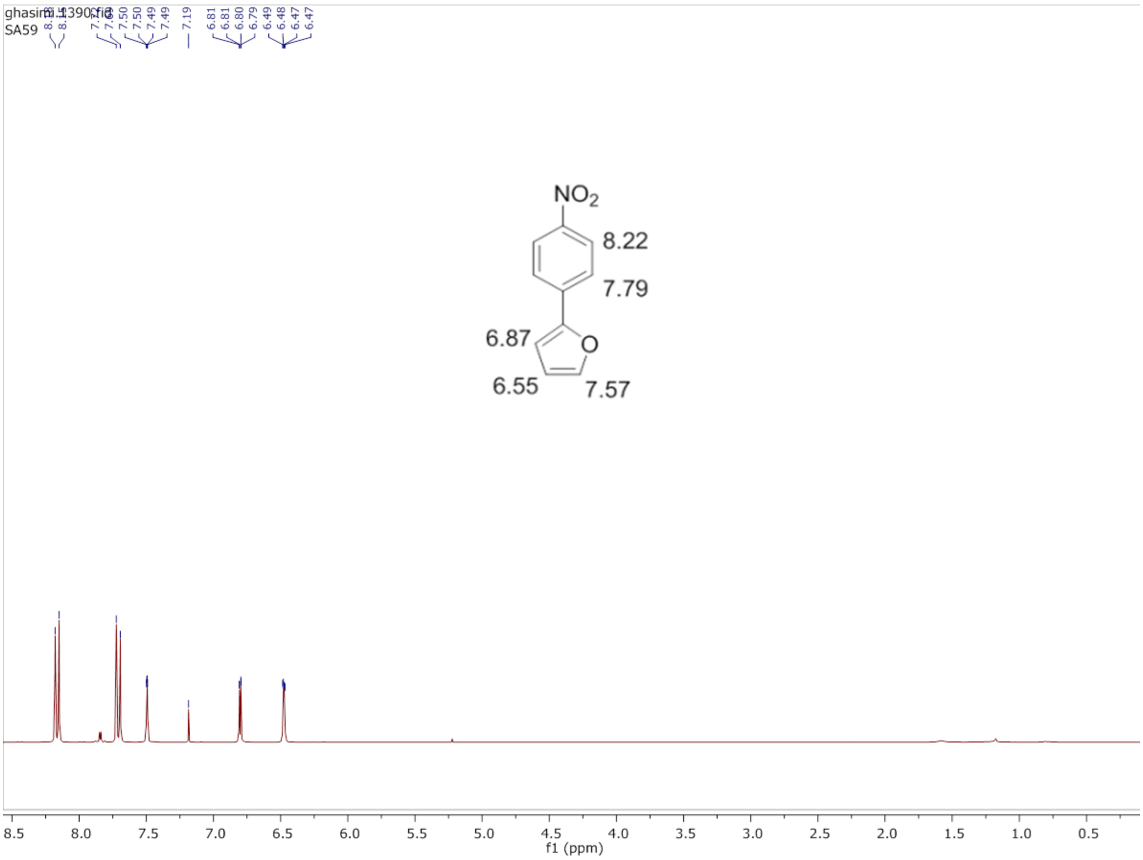

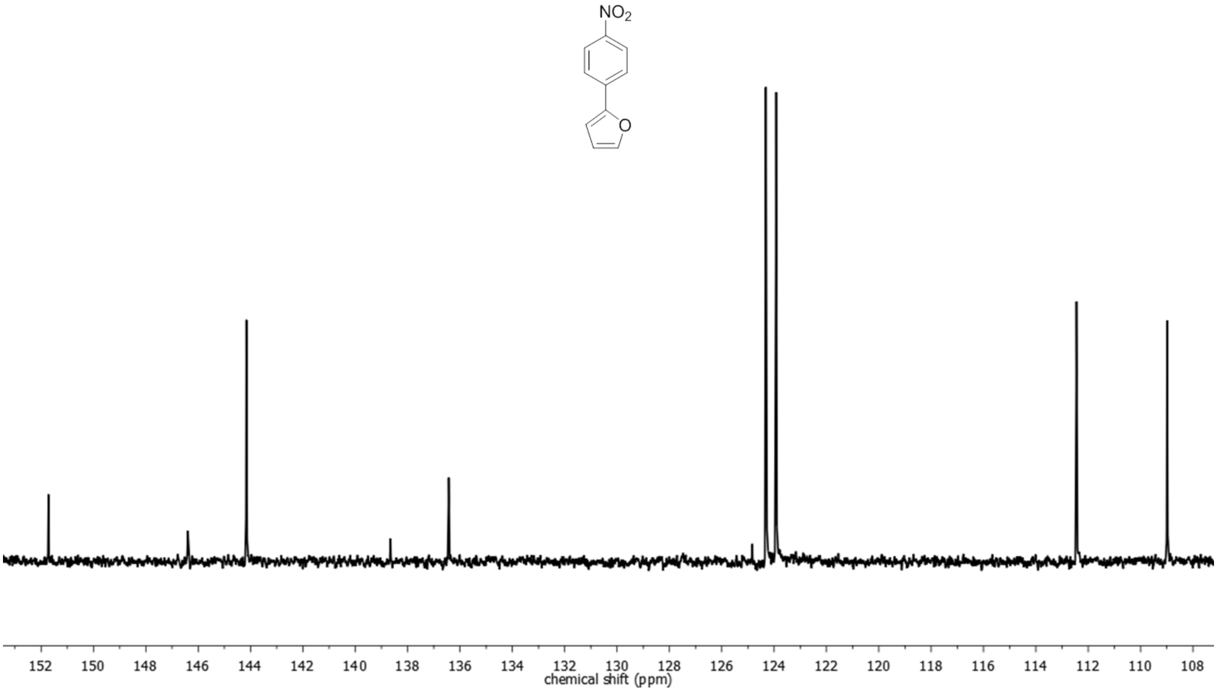

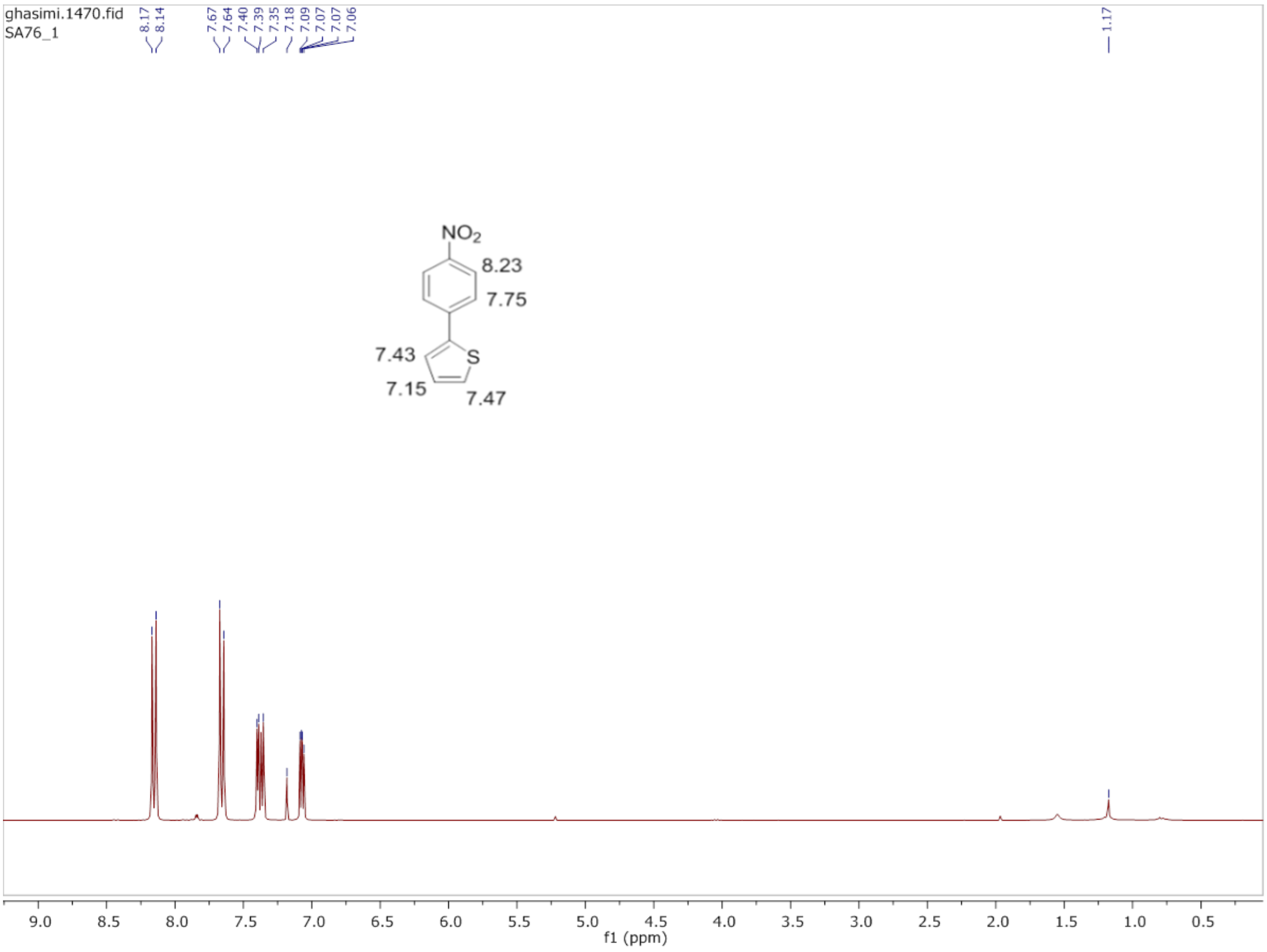
ghasimi.1470.fid
SA76_1
8.17
8.14
7.67
7.64
7.40
7.39
7.35
7.18
7.09
7.07
7.07
7.06
1.17
NO2
8.23
7.75
7.43
S
7.15
7.47
f1 (ppm)

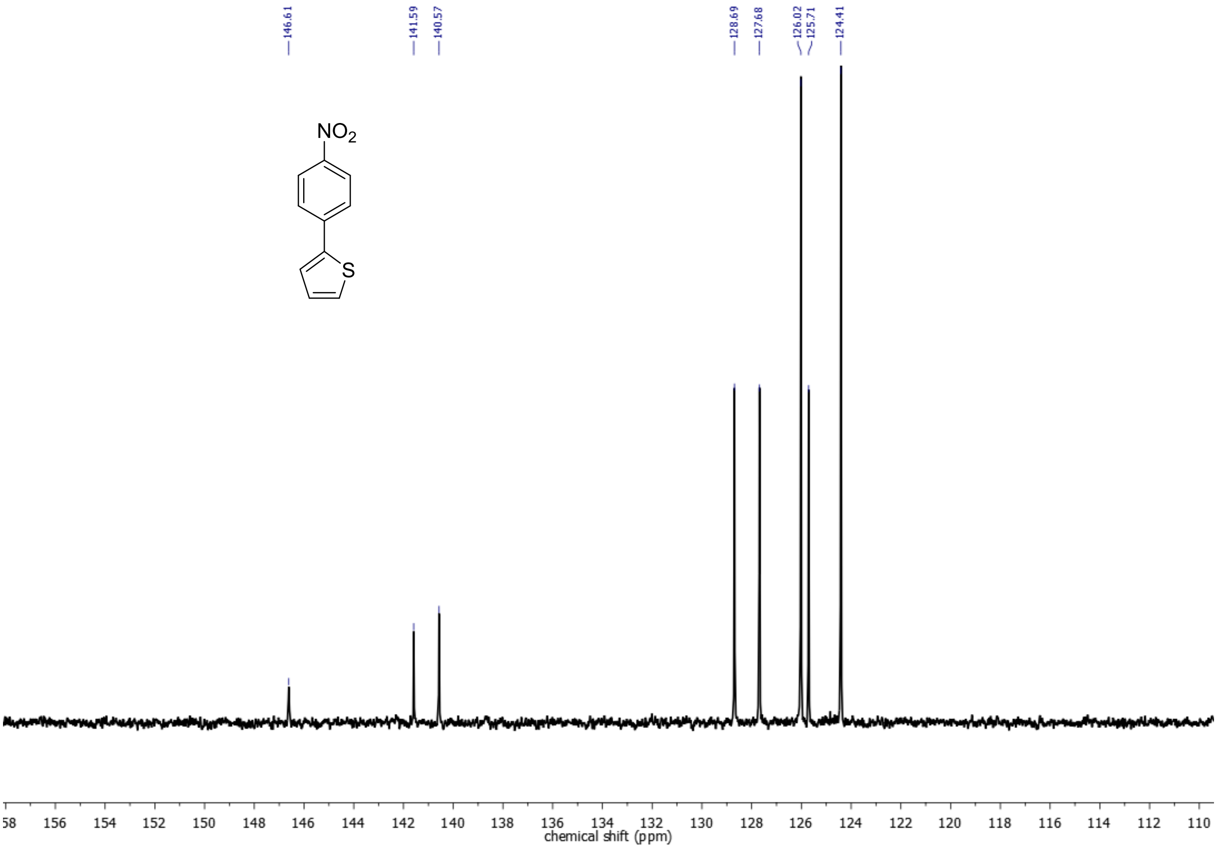
146.61
141.59
140.57
128.69
127.68
126.02
125.71
124.41
NO2
S
chemical shift (ppm)

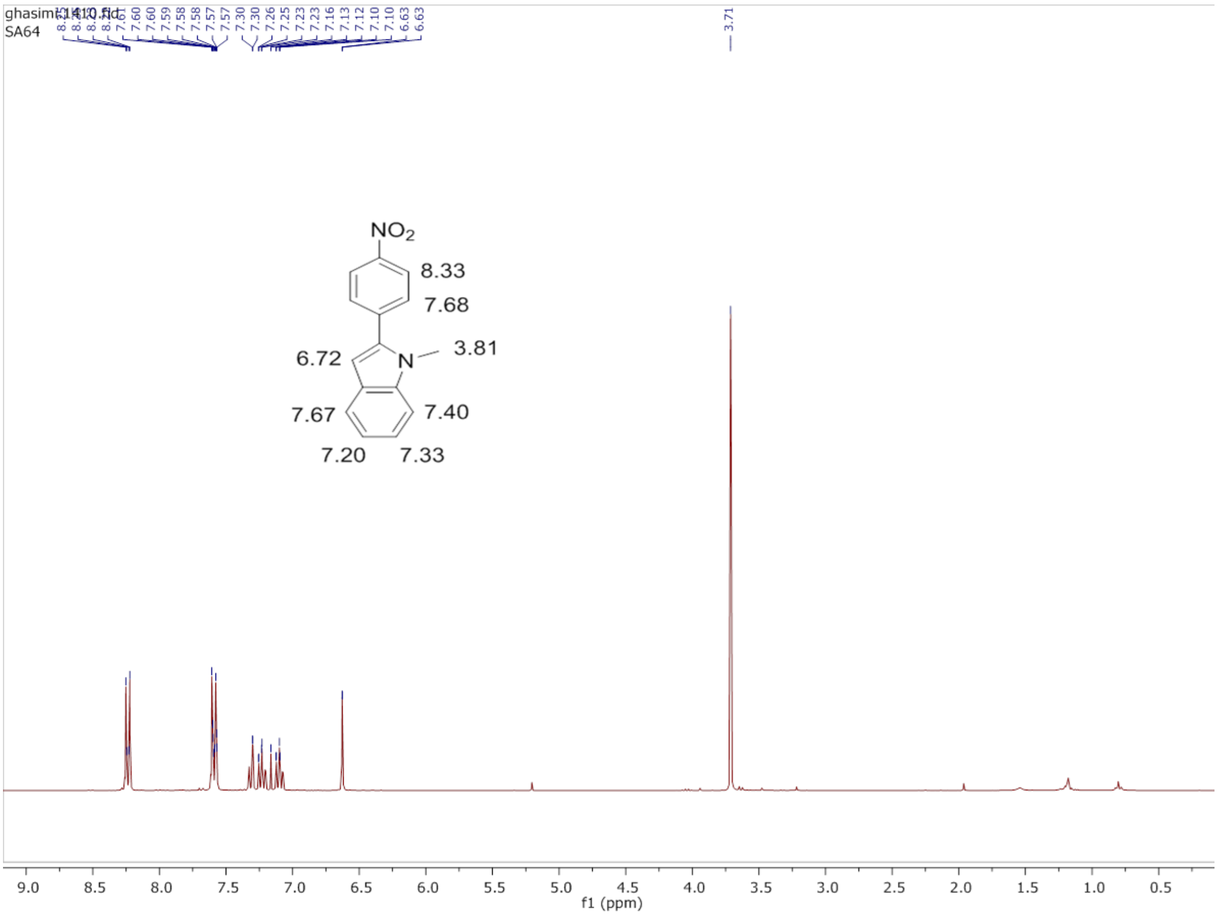
ghasimi-1410.fid
SA64
3.71
8.33
7.68
6.72
3.81
7.67
7.40
7.20
7.33
f1 (ppm)

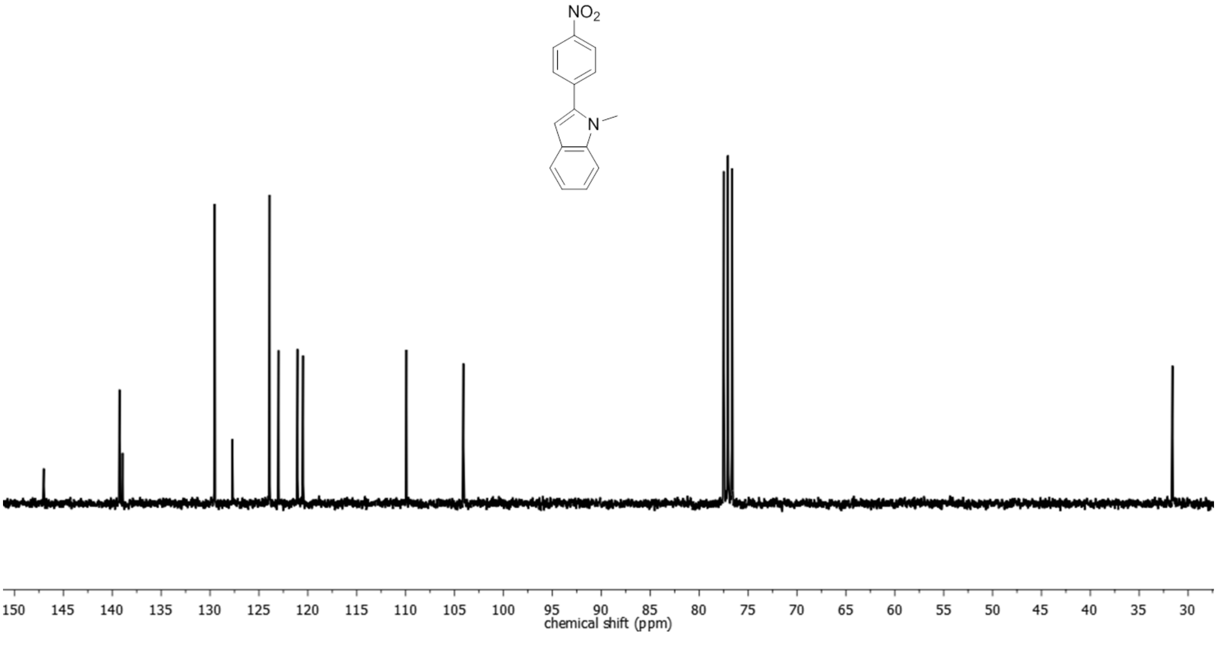
chemical shift (ppm)

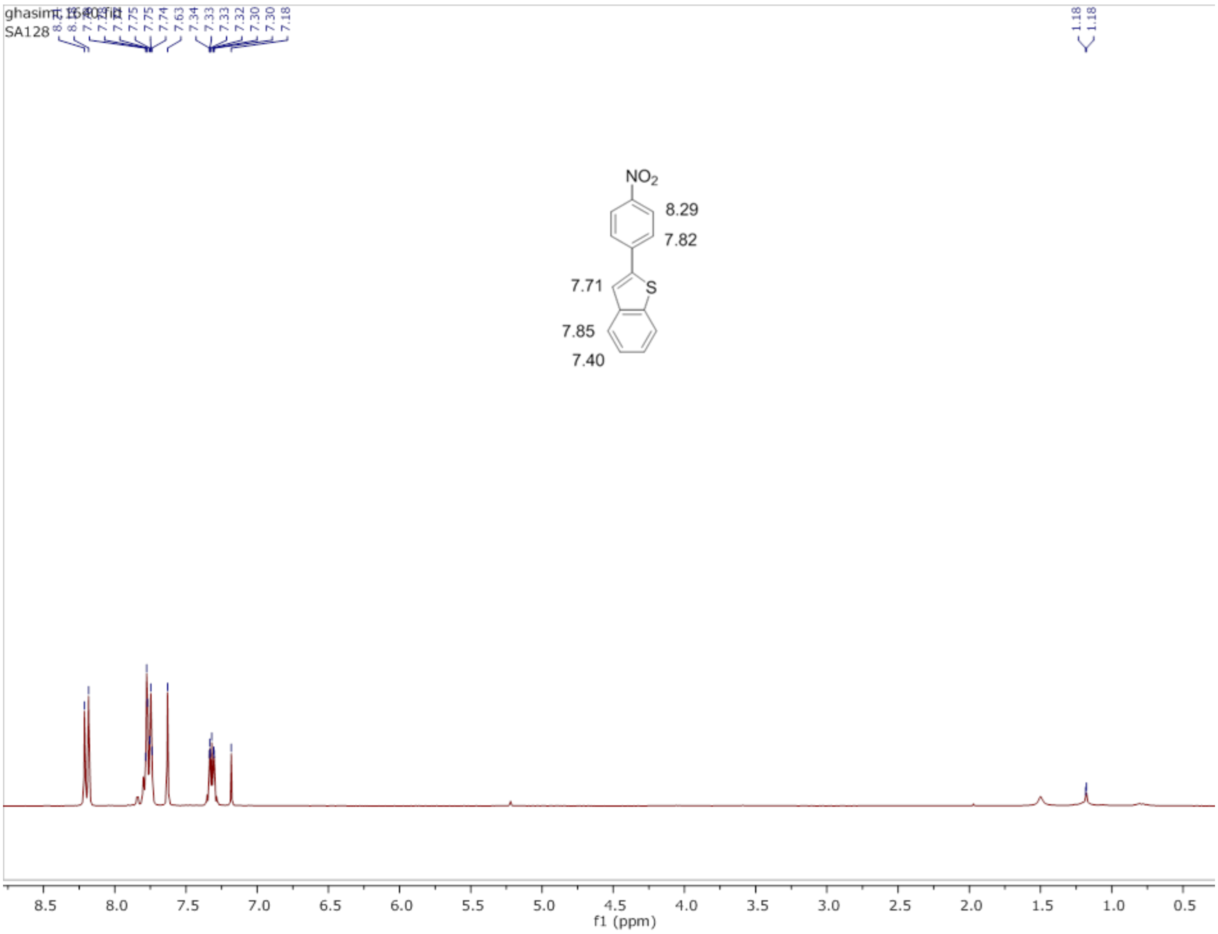
SA128
NO2
8.29
7.82
7.71
S
7.85
7.40
1.18
1.18
f1 (ppm)

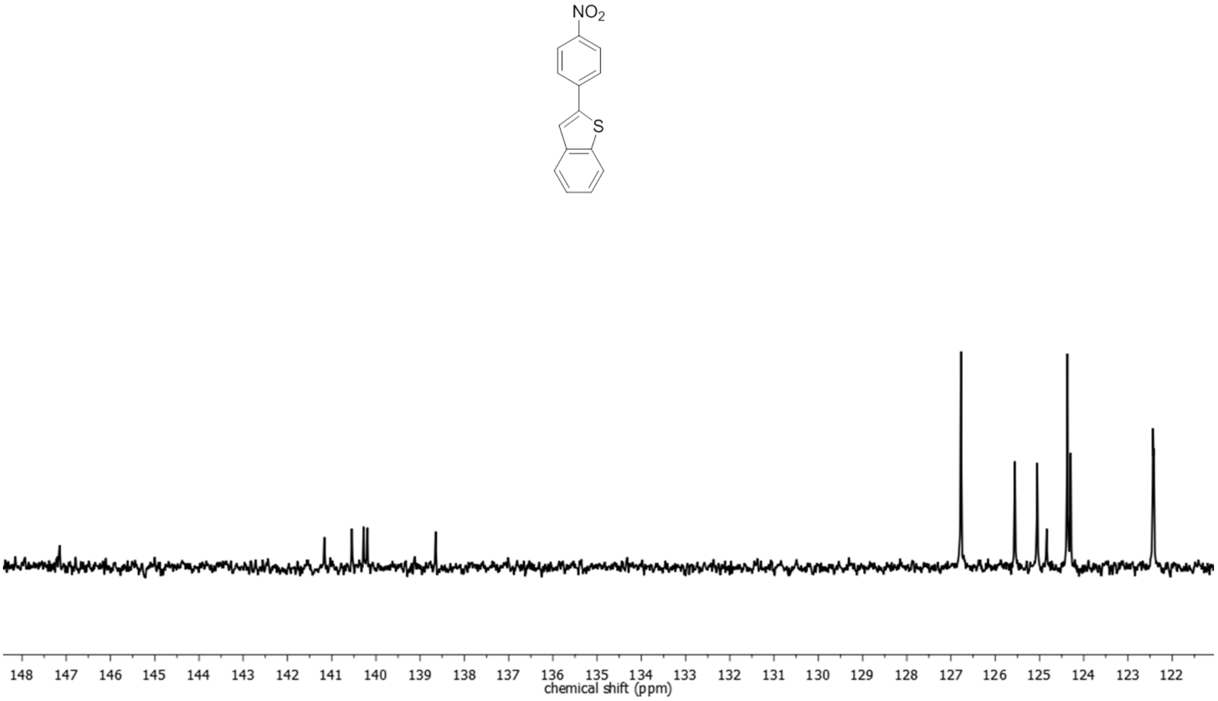
NO2
S
chemical shift (ppm)

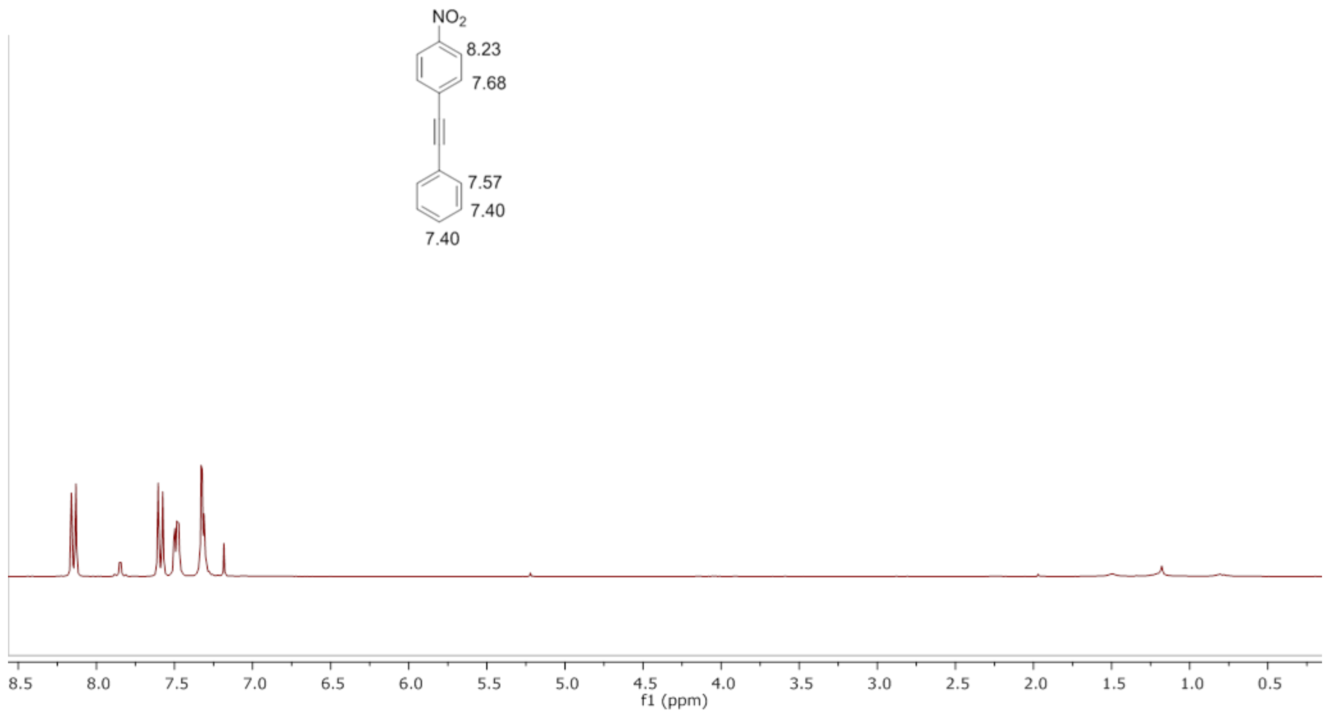
NO2
8.23
7.68
7.57
7.40
7.40
8.5 8.0 7.5 7.0 6.5 6.0 5.5 5.0 4.5 4.0 3.5 3.0 2.5 2.0 1.5 1.0 0.5
f1 (ppm)

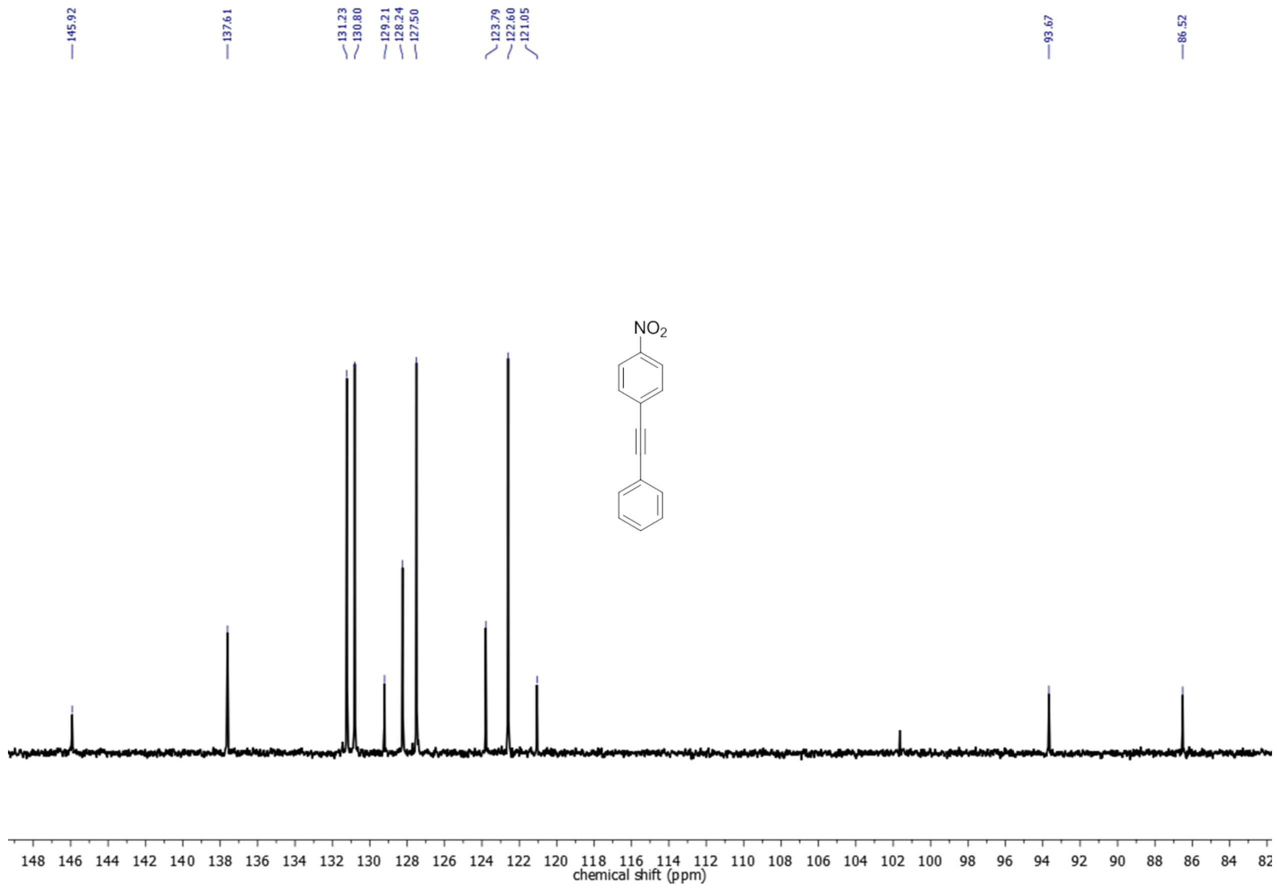
145.92
137.61
131.23
130.80
129.21
128.24
127.50
123.79
122.60
121.05
93.67
86.52
NO2
148 146 144 142 140 138 136 134 132 130 128 126 124 122 120 118 116 114 112 110 108 106 104 102 100 98 96 94 92 90 88 86 84 82
chemical shift (ppm)

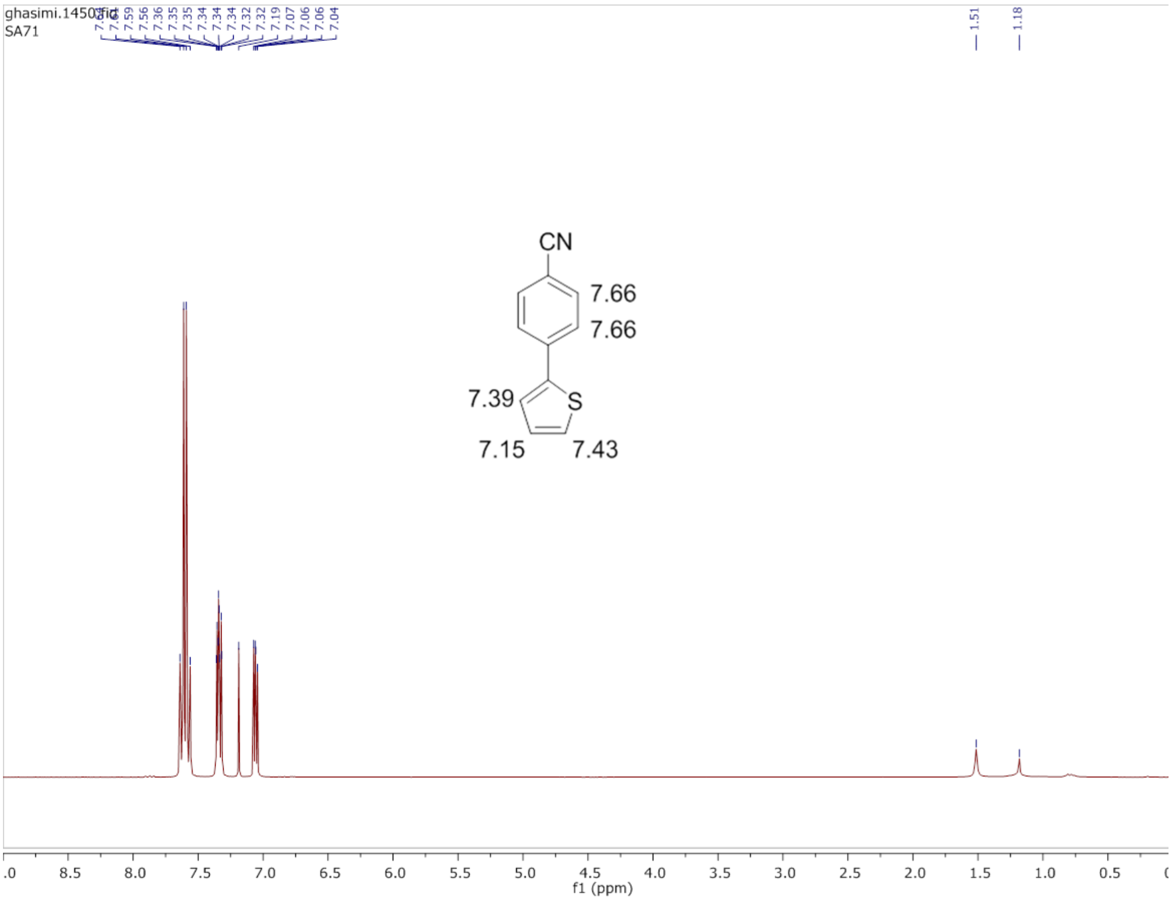
ghasimi.14503.fid
SA71
1.51
1.18
CN
7.66
7.66
7.39
S
7.15
7.43
f1 (ppm)

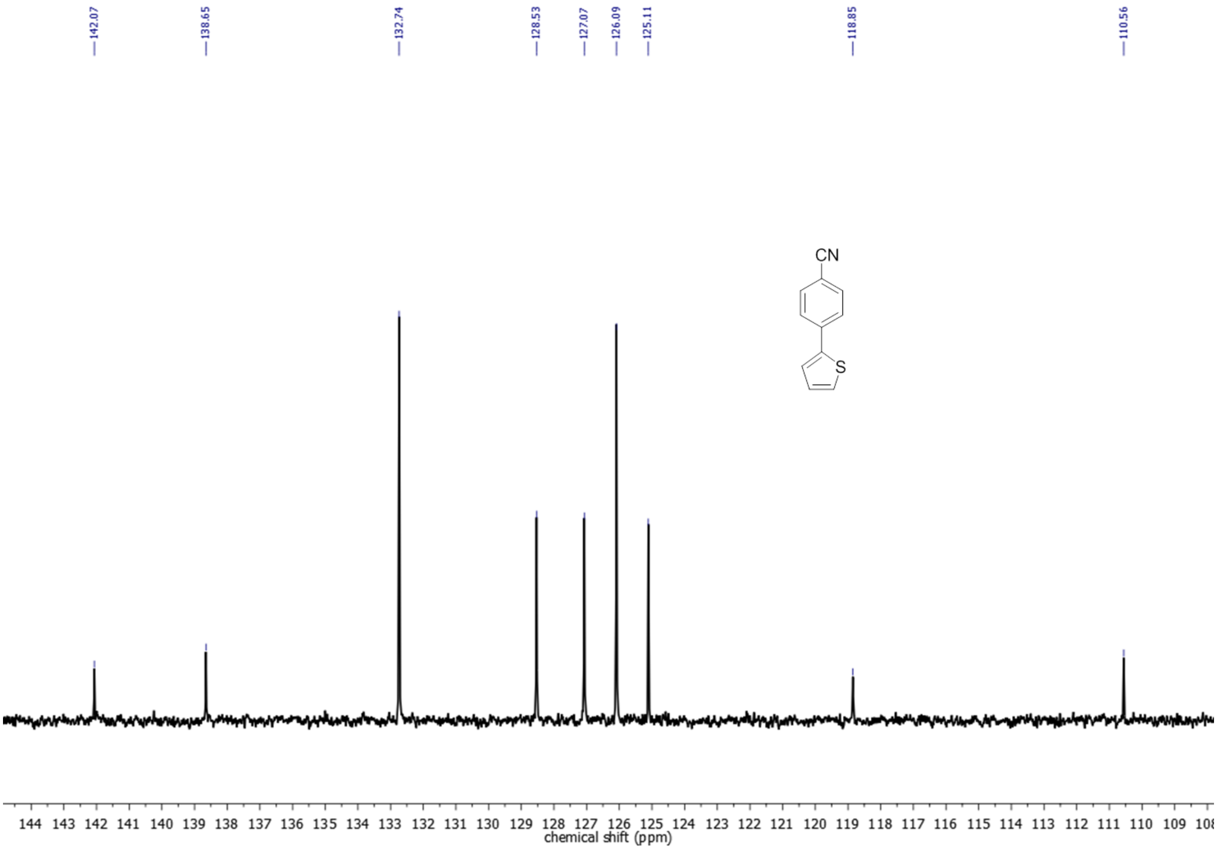
142.07
138.65
132.74
128.53
127.07
126.09
125.11
118.85
110.56
CN
S
chemical shift (ppm)

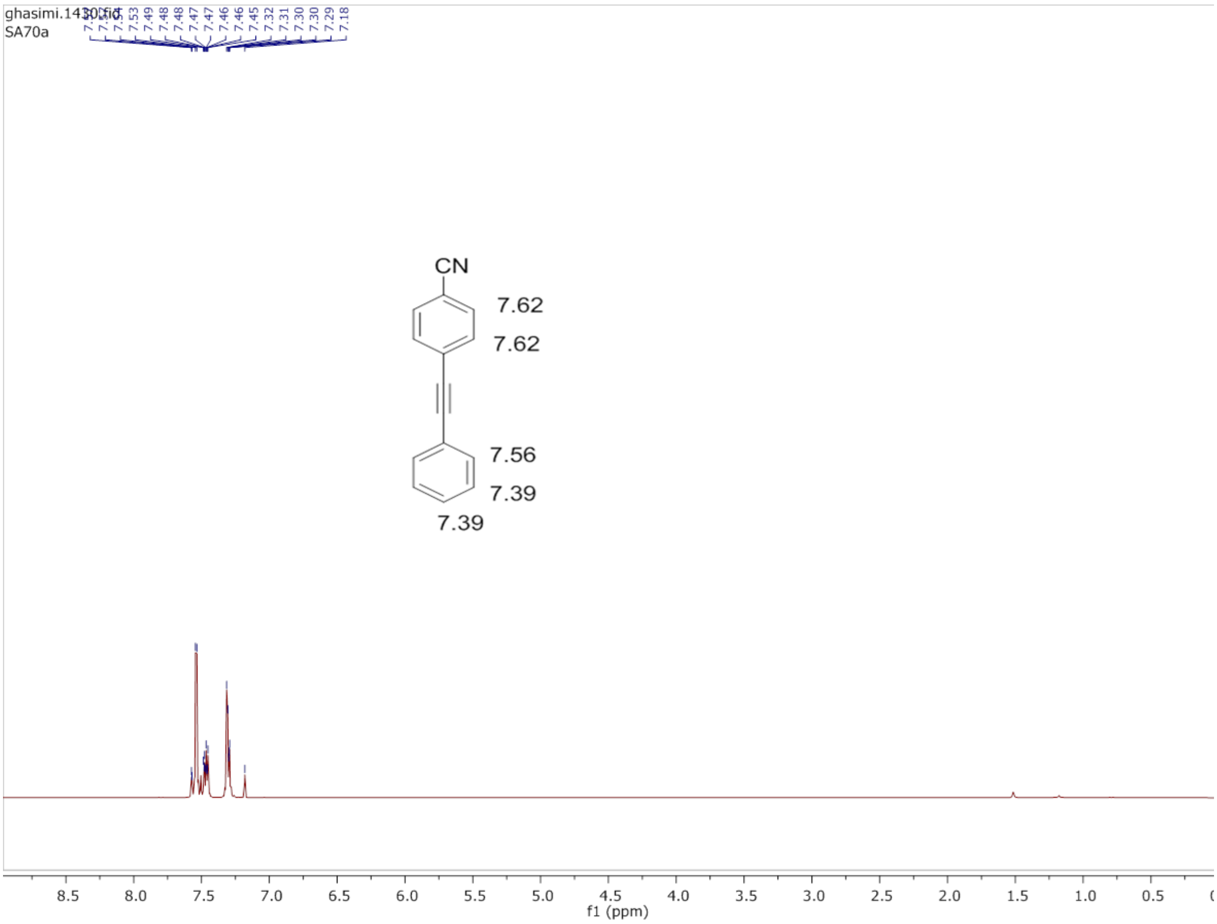
ghasimi.1430.fid
SA70a
CN
7.62
7.62
7.56
7.39
7.39
8.5 8.0 7.5 7.0 6.5 6.0 5.5 5.0 4.5 4.0 3.5 3.0 2.5 2.0 1.5 1.0 0.5
f1 (ppm)

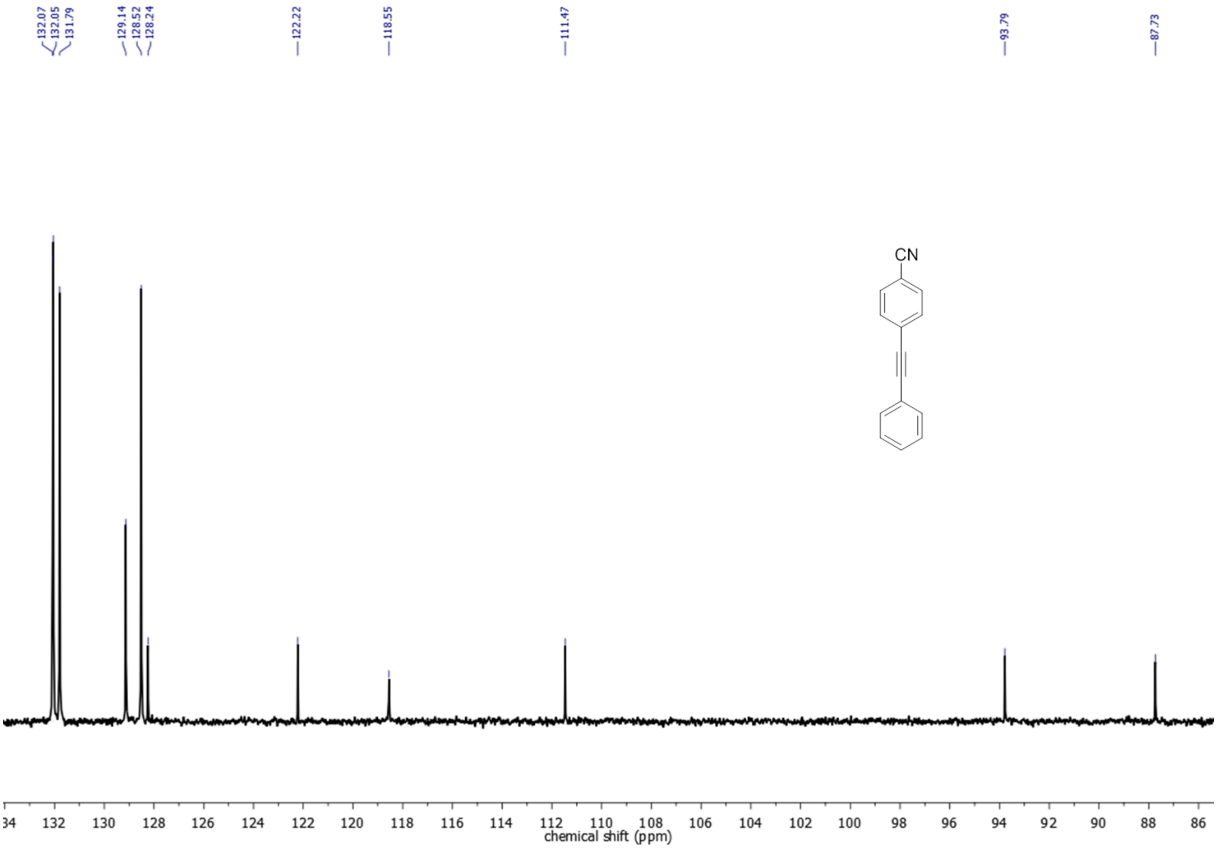
132.07
132.05
131.79
129.14
128.52
128.24
122.22
118.55
111.47
93.79
87.73
CN
132 130 128 126 124 122 120 118 116 114 112 110 108 106 104 102 100 98 96 94 92 90 88 86
chemical shift (ppm)

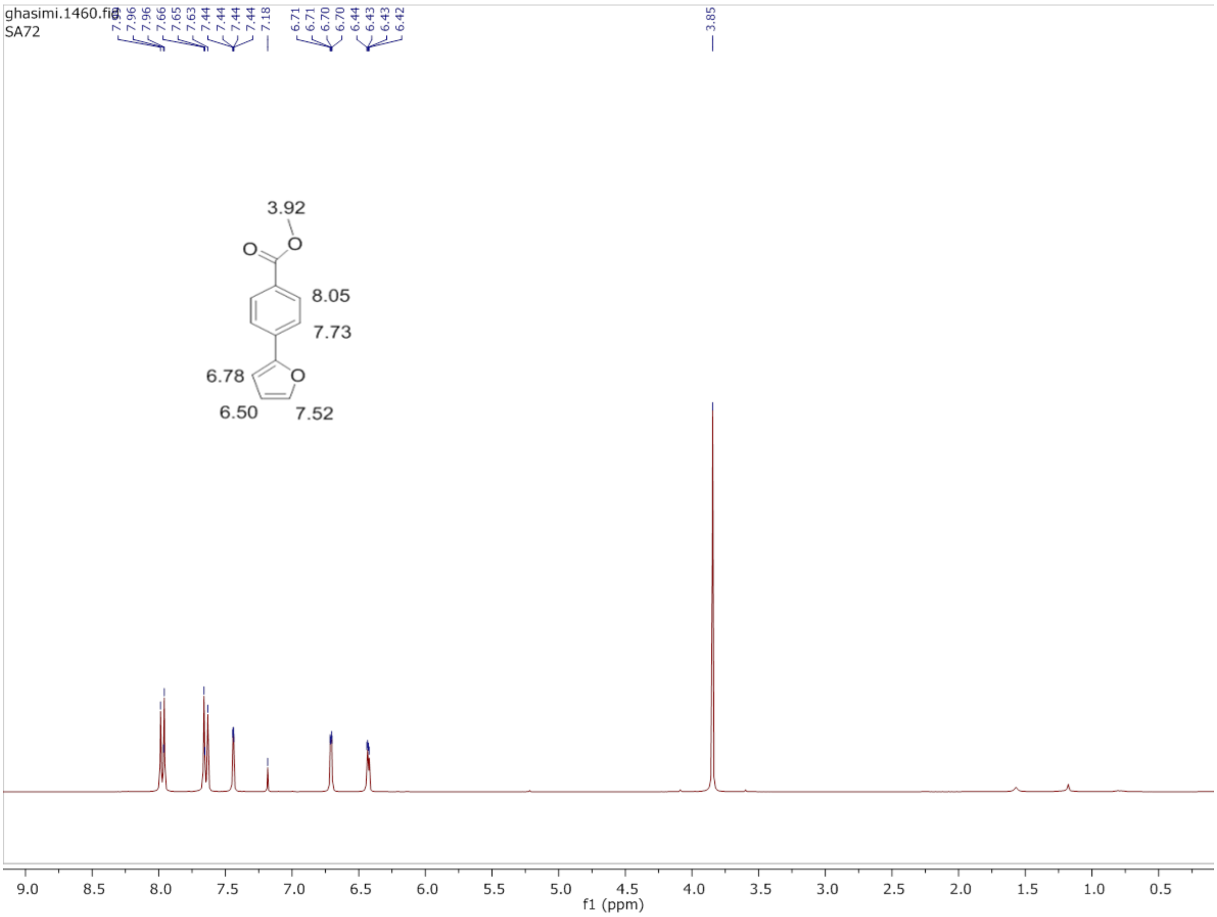
ghasimi.1460.fid
SA72
7.99
7.96
7.96
7.66
7.65
7.63
7.44
7.44
7.44
7.44
7.18
6.71
6.71
6.70
6.70
6.44
6.43
6.43
6.42
3.85
3.92
O
O
8.05
7.73
6.78
O
6.50
7.52
9.0
8.5
8.0
7.5
7.0
6.5
6.0
5.5
5.0
4.5
4.0
3.5
3.0
2.5
2.0
1.5
1.0
0.5
f1 (ppm)

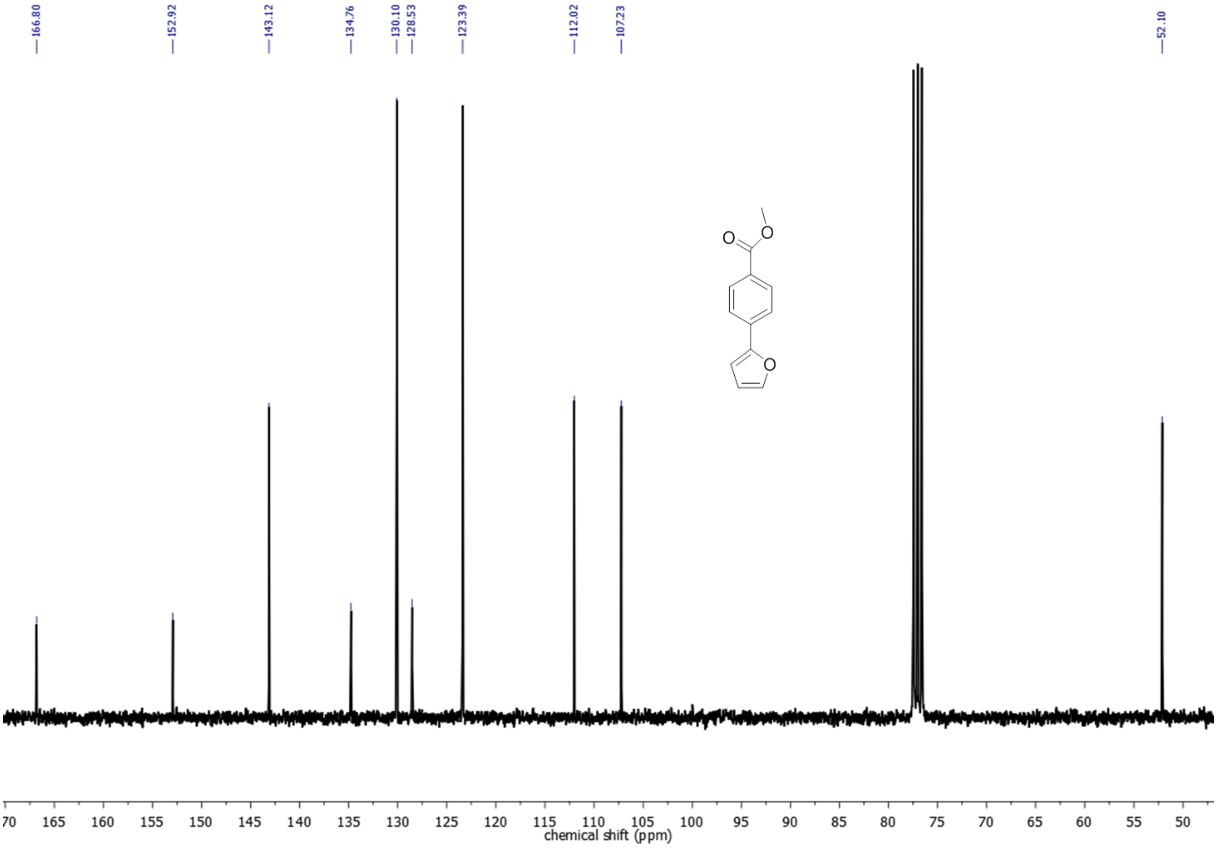
166.80
152.92
143.12
134.76
130.10
128.53
123.39
112.02
107.23
52.10
O
O
O
70
165
160
155
150
145
140
135
130
125
120
115
110
105
100
95
90
85
80
75
70
65
60
55
50
chemical shift (ppm)

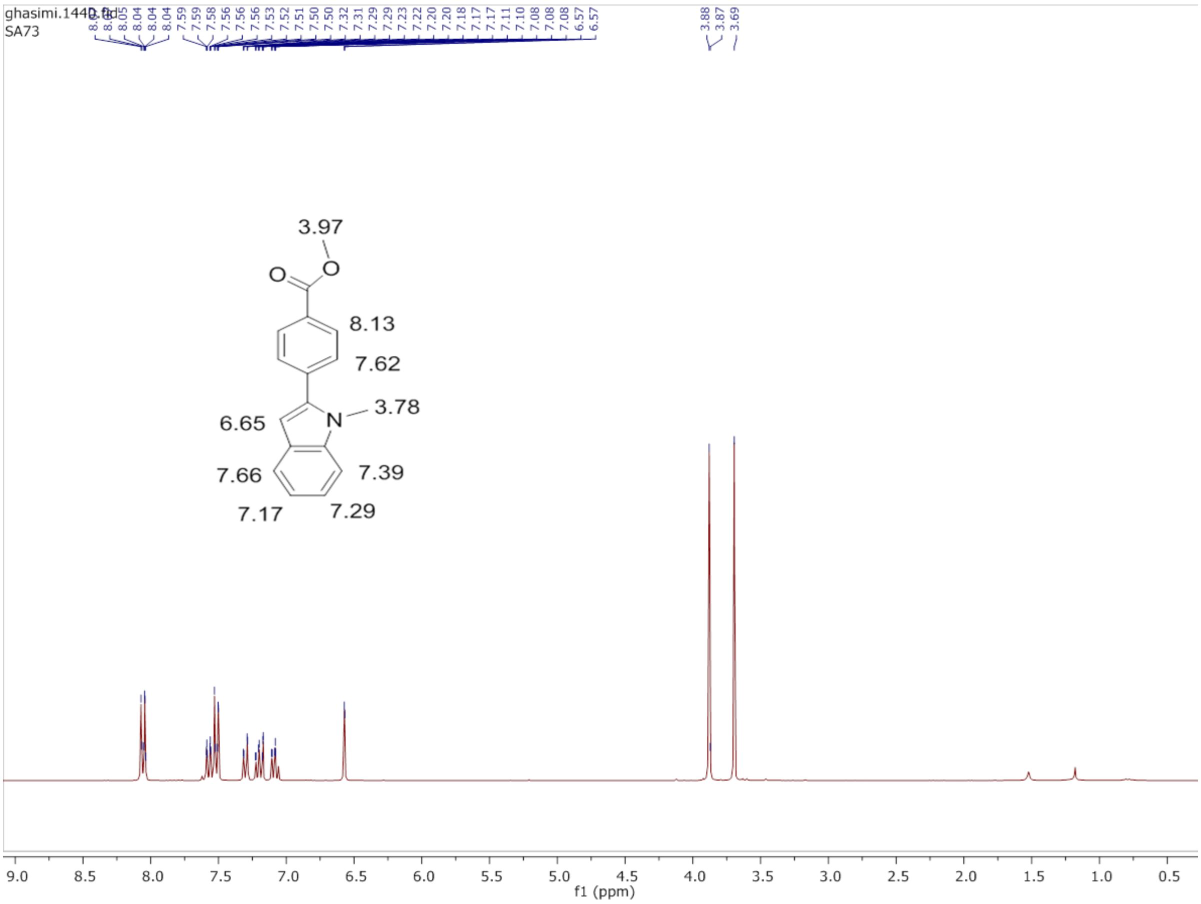
ghasimi.1440.fid
SA73
3.97
O
O
8.13
7.62
N
3.78
6.65
7.66
7.39
7.17
7.29
3.88
3.87
3.69
9.0
8.5
8.0
7.5
7.0
6.5
6.0
5.5
5.0
4.5
4.0
3.5
3.0
2.5
2.0
1.5
1.0
0.5
f1 (ppm)

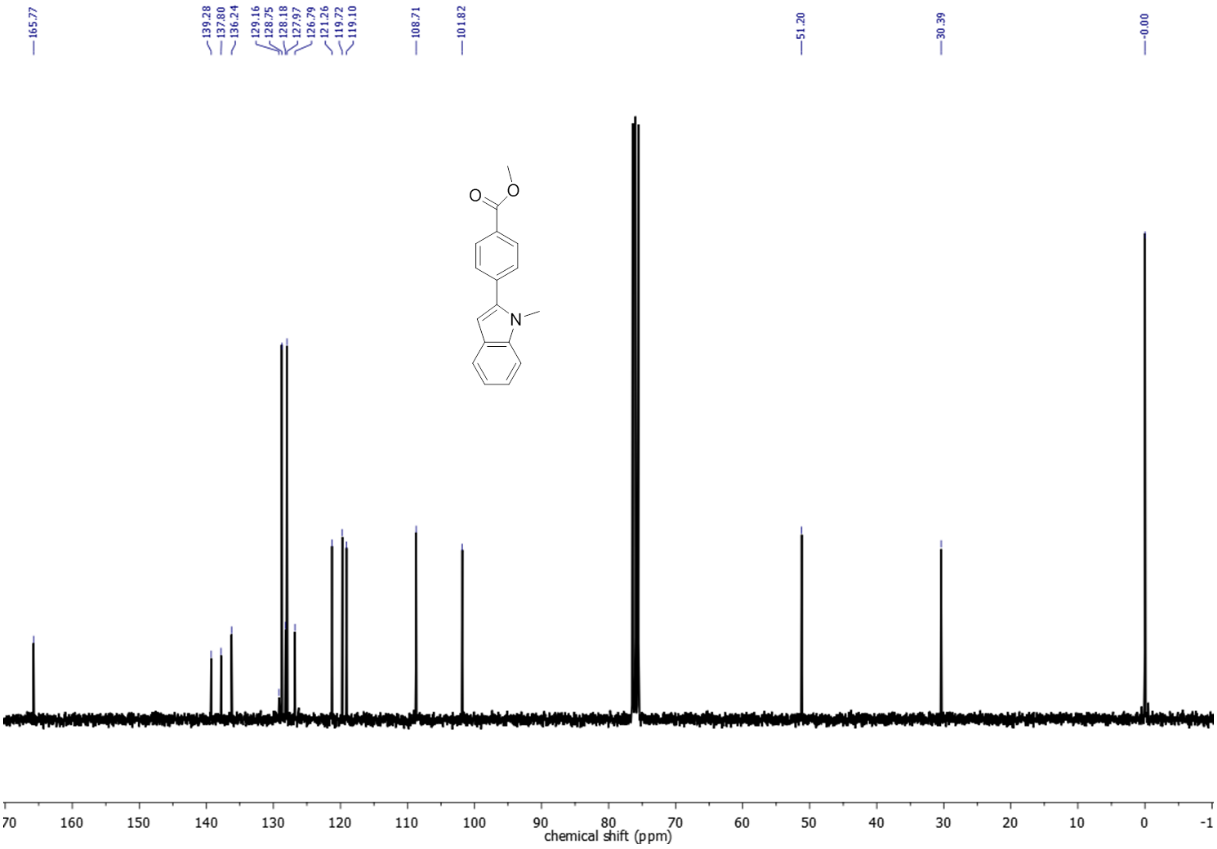
165.77
139.28
137.80
136.24
129.16
128.75
128.18
127.97
126.79
121.26
119.72
119.10
108.71
101.82
51.20
30.39
-0.00
O
O
N
chemical shift (ppm)

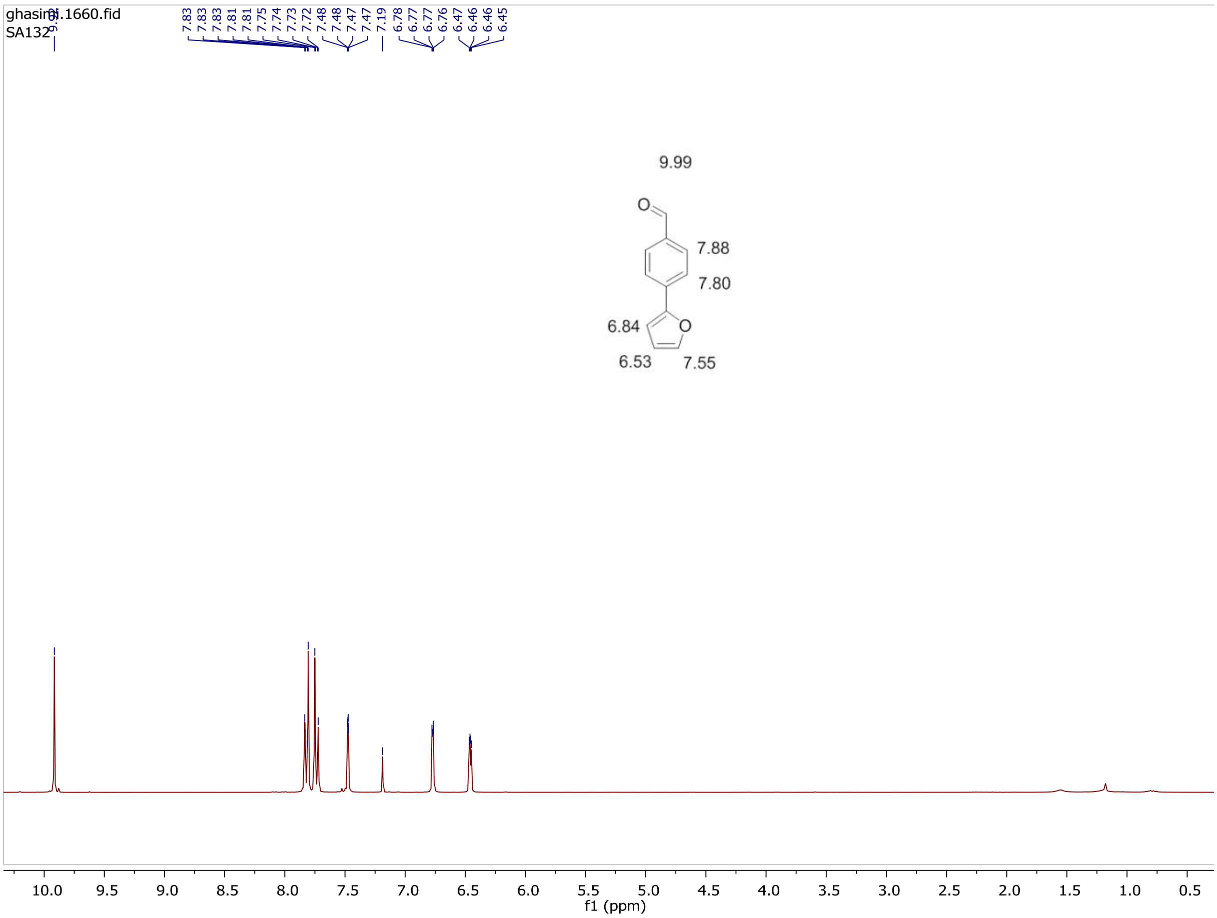
ghasimi.1660.fid
SA132
7.83
7.83
7.83
7.81
7.81
7.75
7.74
7.73
7.72
7.48
7.47
7.47
7.19
6.78
6.77
6.76
6.47
6.46
6.46
6.45
9.99
O
7.88
7.80
6.84
O
6.53
7.55
10.0
9.5
9.0
8.5
8.0
7.5
7.0
6.5
6.0
5.5
5.0
4.5
4.0
3.5
3.0
2.5
2.0
1.5
1.0
0.5
f1 (ppm)

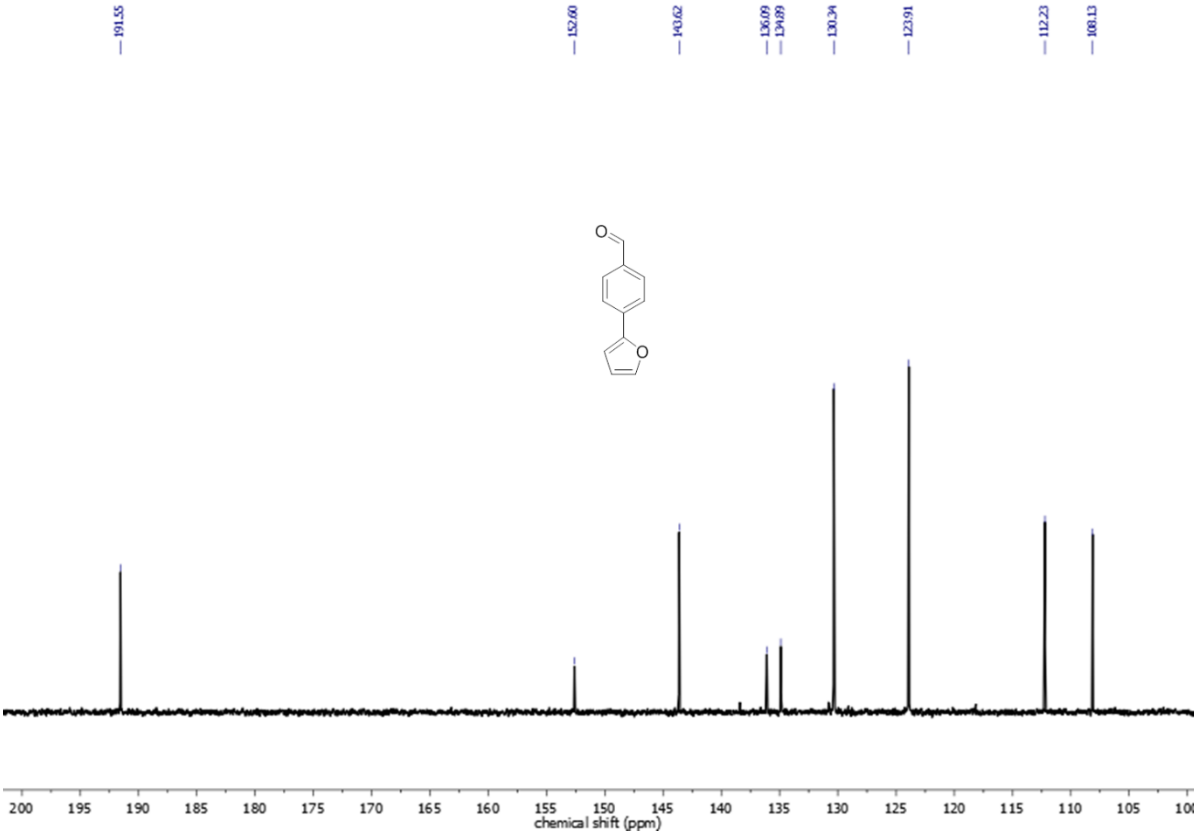
191.55
152.60
143.62
136.09
134.89
130.34
123.91
112.23
108.13
O
O
200
195
190
185
180
175
170
165
160
155
150
145
140
135
130
125
120
115
110
105
chemical shift (ppm)

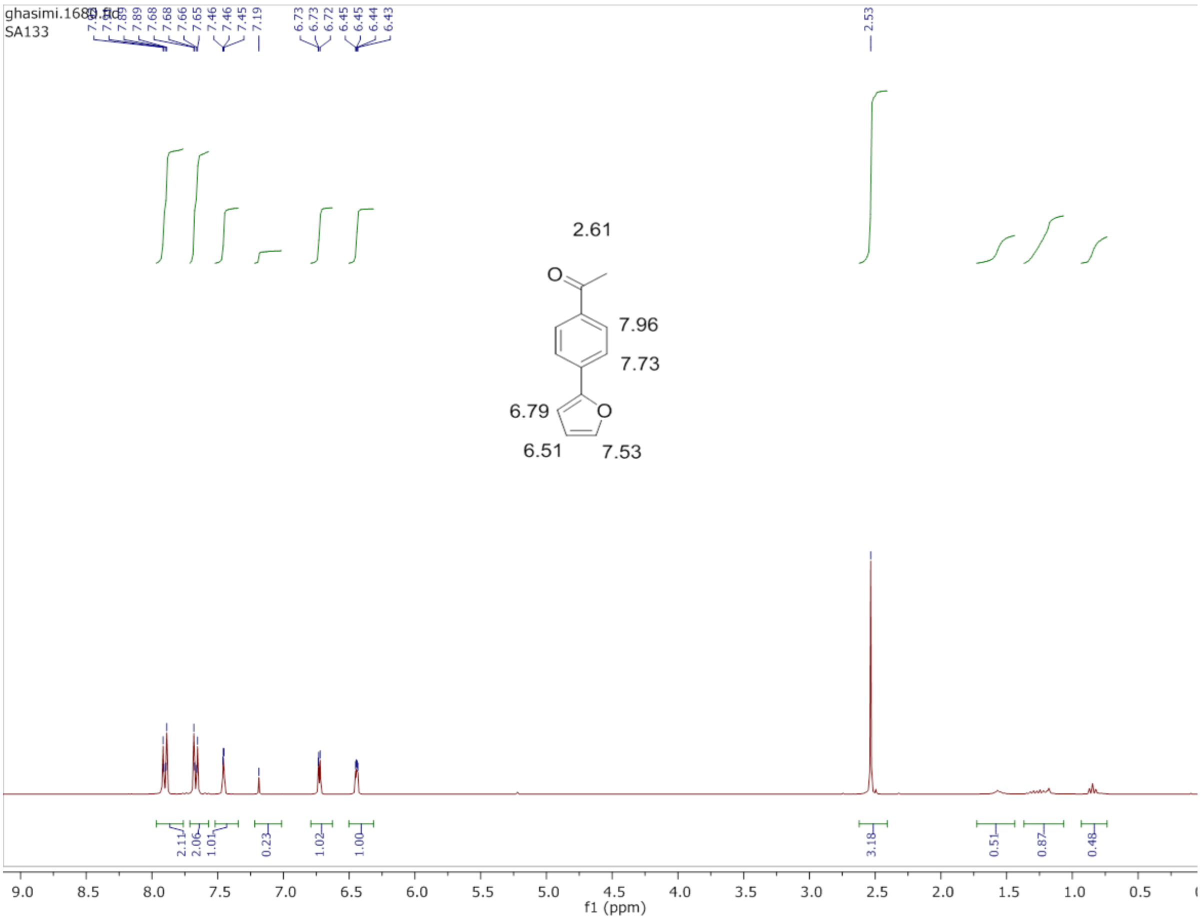
ghasimi.1680.fid
SA133
2.53
2.61
7.96
7.73
6.79
6.51
7.53
2.11
2.06
1.01
0.23
1.02
1.00
3.18
0.51
0.87
0.48
f1 (ppm)

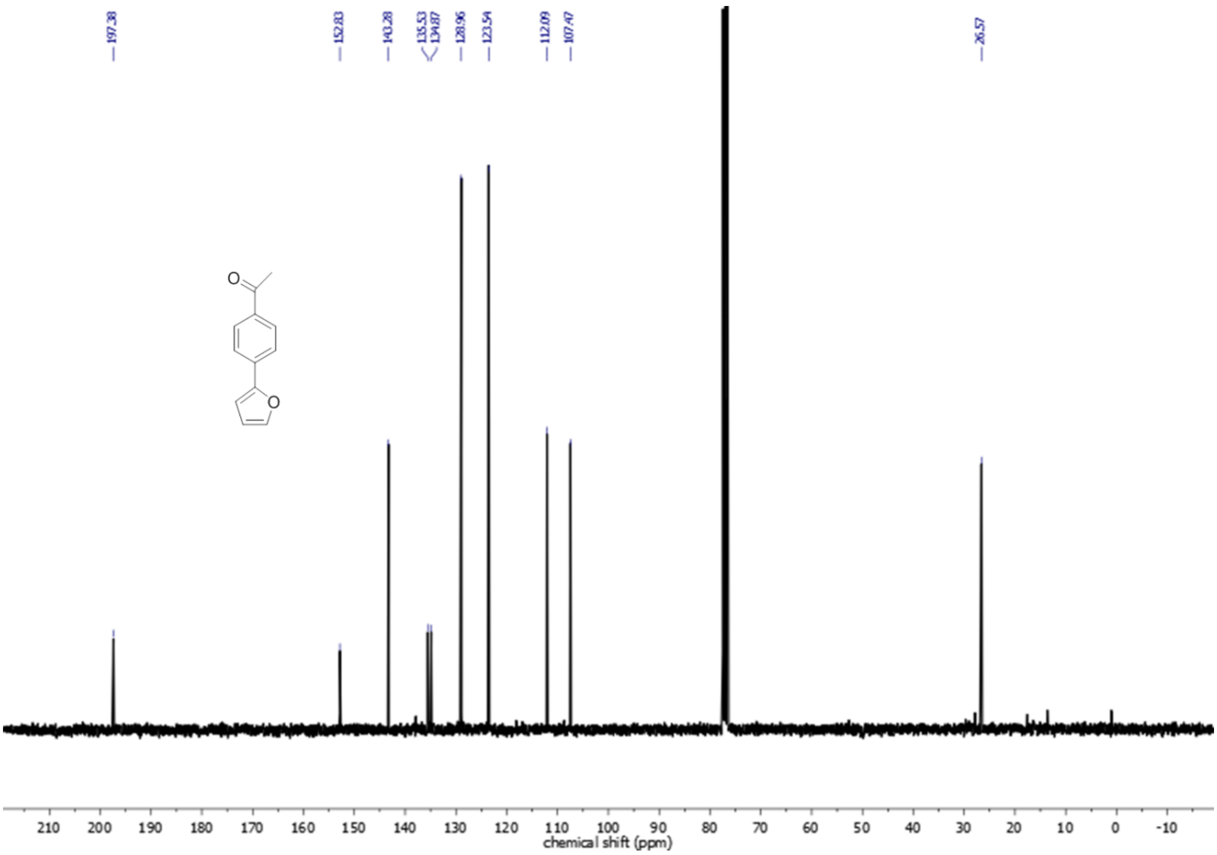
197.38
152.83
143.28
135.53
134.87
128.96
123.54
112.09
107.47
26.57
chemical shift (ppm)

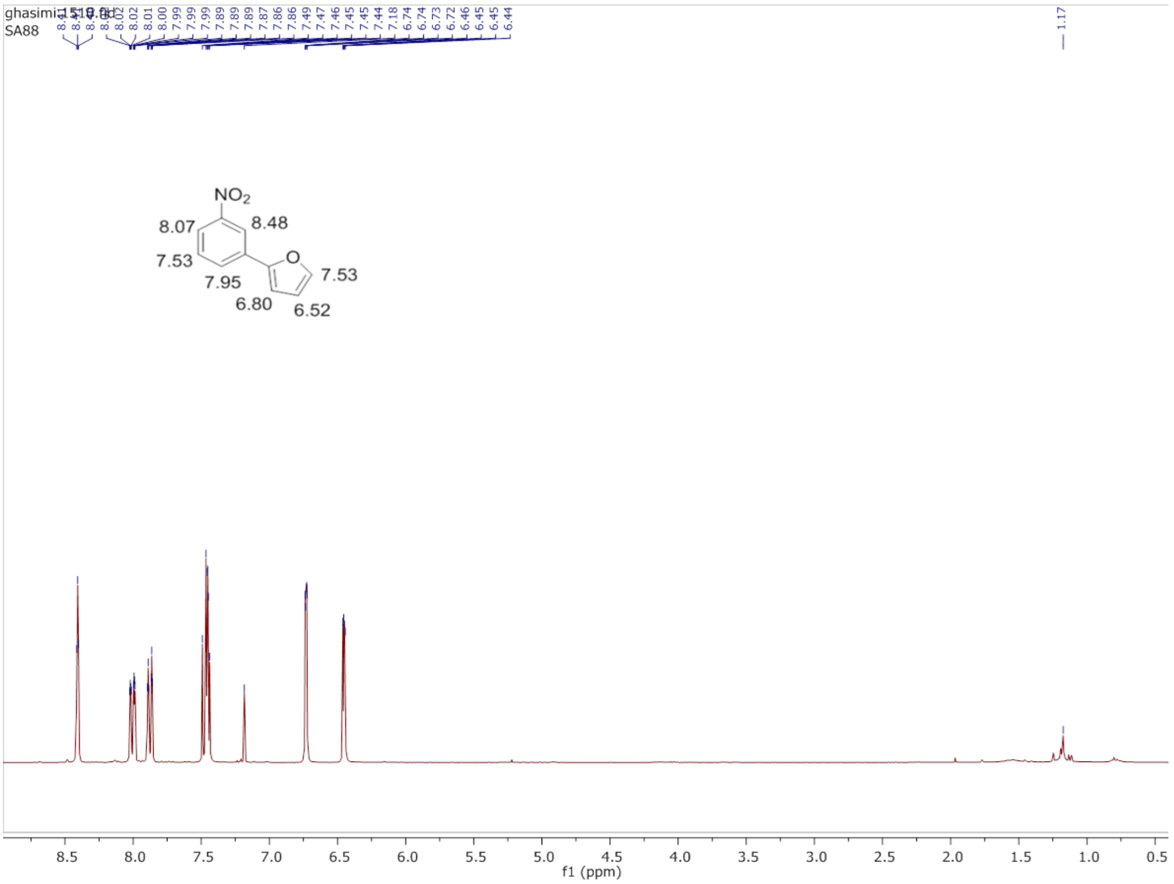
ghasimi-1510.fid
SA88
1.17
NO2
8.07
8.48
7.53
7.95
7.53
6.80
6.52
8.5 8.0 7.5 7.0 6.5 6.0 5.5 5.0 4.5 4.0 3.5 3.0 2.5 2.0 1.5 1.0 0.5
f1 (ppm)

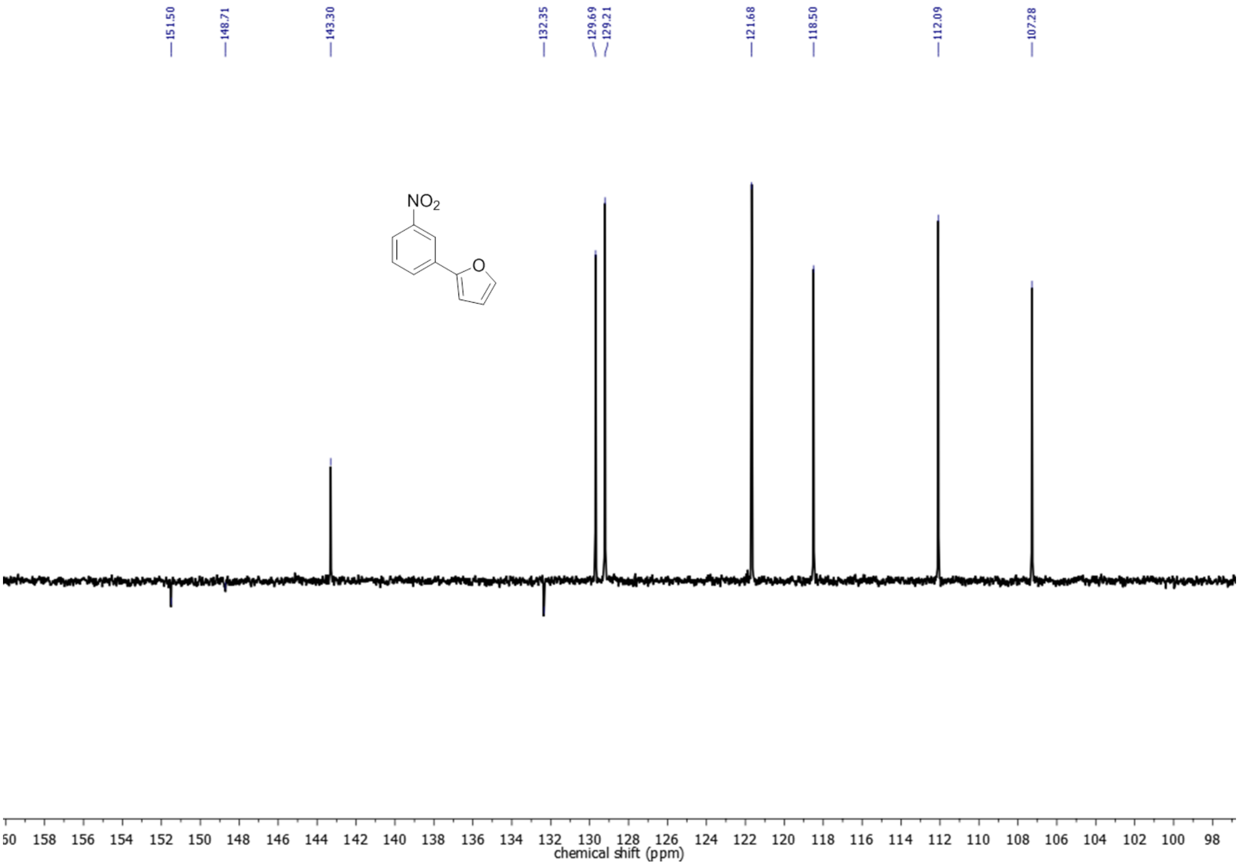
151.50
148.71
143.30
132.35
129.69
129.21
121.68
118.50
112.09
107.28
NO2
chemical shift (ppm)

(1) Brouwer, F.; Alma, J.; Valkenier, H.; Voortman, T. P.; Hillebrand, J.; Chiechi, R. C.; Hummelen, J. C. *J. Mater. Chem.* **2011**, *21*, 1582-1592.

(2) Frisch, M. J.; Trucks, G. W.; Schlegel, H. B.; Scuseria, G. E.; Robb, M. A.; Cheeseman, J. R.; Scalmani, G.; Barone, V.; Mennucci, B.; Petersson, G. A.; Nakatsuji, H.; Caricato, M.; Li, X.; Hratchian, H. P.; Izmaylov, A. F.; Bloino, J.; Zheng, G.; Sonnenberg, J. L.; Hada, M.; Ehara, M.; Toyota, K.; Fukuda, R.; Hasegawa, J.; Ishida, M.; Nakajima, T.; Honda, Y.; Kitao, O.; Nakai, H.; Vreven, T.; Montgomery Jr., J. A.; Peralta, J. E.; Ogliaro, F.; Bearpark, M. J.; Heyd, J.; Brothers, E. N.; Kudin, K. N.; Staroverov, V. N.; Kobayashi, R.; Normand, J.; Raghavachari, K.; Rendell, A. P.; Burant, J. C.; Iyengar, S. S.; Tomasi, J.; Cossi, M.; Rega, N.; Millam, N. J.; Klene, M.; Knox, J. E.; Cross, J. B.; Bakken, V.; Adamo, C.; Jaramillo, J.; Gomperts, R.; Stratmann, R. E.; Yazyev, O.; Austin, A. J.; Cammi, R.; Pomelli, C.; Ochterski, J. W.; Martin, R. L.; Morokuma, K.; Zakrzewski, V. G.; Voth, G. A.; Salvador, P.; Dannenberg, J. J.; Dapprich, S.; Daniels, A. D.; Farkas, Ö.; Foresman, J. B.; Ortiz, J. V.; Cioslowski, J.; Fox, D. J.; Gaussian, Inc.: Wallingford, CT, USA, 2009.

(3) McNaught, A. D., Wilkinson, A. In *International Union of Pure and Applied Chemistry (IUPAC) Gold Book*; 2nd ed.; Blackwell Science: Oxford UK.
